# Contribution of farms to the microbiota in the swine value chain

**DOI:** 10.3389/fsysb.2023.1183868

**Published:** 2023-07-12

**Authors:** Pascal Laforge, Antony T. Vincent, Caroline Duchaine, Perrine Feutry, Annick Dion-Fortier, Pier-Luc Plante, Éric Pouliot, Sylvain Fournaise, Linda Saucier

**Affiliations:** ^1^ Département des sciences animales, Université Laval, Québec, QC, Canada; ^2^ Institut sur la nutrition et les aliments fonctionnels, Université Laval, Québec, QC, Canada; ^3^ Centre de recherche en infectiologie porcine et avicole, Faculté de médecine vétérinaire, Université de Montréal, Saint-Hyacinthe, QC, Canada; ^4^ Département de biochimie, microbiologie et bio-Informatique, Université Laval, Québec, QC, Canada; ^5^ Olymel S.E.C./L.P., Boucherville, QC, Canada

**Keywords:** sanitary status, swine value chain, food safety, 16S rDNA, microbiota, microbial ecology, bacteria, meat

## Abstract

**Introduction:** A thorough understanding of the microbial ecology within the swine value chain is essential to develop new strategies to optimize the microbiological quality of pork products. To our knowledge, no study to date has followed the microbiota through the value chain from live farm animals to the cuts of meat obtained for market. The objective of this study is to evaluate how the microbiota of pigs and their environment influence the microbial composition of samples collected throughout the value chain, including the meat plant and meat cuts.

**Method and results:** Results from 16S rDNA sequencing, short-chain fatty acid concentrations and metabolomic analysis of pig feces revealed that the microbiota from two farms with differing sanitary statuses were distinctive. The total aerobic mesophilic bacteria and *Enterobacteriaceae* counts from samples collected at the meat plant after the pre-operation cleaning and disinfection steps were at or around the detection limit and the pigs from the selected farms were the first to be slaughtered on each shipment days. The bacterial counts of individual samples collected at the meat plant did not vary significantly between the farms. Alpha diversity results indicate that as we move through the steps in the value chain, there is a clear reduction in the diversity of the microbiota. A beta diversity analysis revealed a more distinct microbiota at the farms compared to the meat plant which change and became more uniform as samples were taken towards the end of the value chain. The source tracker analysis showed that only 12.92% of the microbiota in shoulder samples originated from the farms and 81% of the bacteria detected on the dressed carcasses were of unknown origin.

**Discussion:** Overall, the results suggest that with the current level of microbial control at farms, it is possible to obtain pork products with similar microbiological quality from different farms. However, broader studies are required to determine the impact of the sanitary status of the herd on the final products.

## 1 Introduction

Pork makes up a major portion of global meat consumption and up until 2015, it was the world’s most consumed meat (OECD, 2021). Like all muscles from healthy animals, with the exception of lymph nodes, pork harbours a small number of microorganisms (Huffman, 2002). Meat and meat products are often identified as causing foodborne illnesses (Painter et al., 2013; Ramsay and Delisle, 2017). Meat is rich in nutrients and water, which supports microbial growth (Remenant et al., 2015; Zagorec and Champomier-Vergès, 2017). As such, contamination of swine carcasses and the resulting cuts of meat can lead to spoilage or pathogen growth throughout the shelf life of the product. Contamination of the meat can occur through microorganisms that are present in different parts of the animal (digestive tract, skin, respiratory tract, saliva, etc.; Zweifel and Stephan, 2014). Hence, pork quality relies on a combination of effective biosecurity measures, herd health management at the farm, hygienic slaughter and cutting practices and risk management measures throughout the value chain (HACCP plans and similar measures).

The sanitary status or health status of a farm is determined by a veterinarian through the monitoring of current and historical diseases as well as the current sanitation conditions of the farm. This status is thought to influence the final microbial quality of the product (Hurd et al., 2008; Vigors et al., 2020). Proper health management and other sanitary measures, including on-farm biosecurity, reduce the risk of infection from major swine pathogens such as pathogenic serovars of *Mycoplasma hyopneumoniae* (enzootic pneumonia) and *Actinobacillus pleuropneumoniae* (pleuropneumonia; Stärk et al., 2008). Other pathogens, including *Clostridium perfringens*, *Salmonella enterica* and *Yersinia enterocolitica* have been linked with carcass contamination (Fosse et al., 2008). While modern pig farms generally maintain excellent sanitary status, there is a certain level of variability between farms (Cameron, 2000). Improving the sanitary conditions at the farm can also improve animal health and behaviour (Meer et al., 2017). To properly evaluate the impact of the sanitary status of farms on the microbiota throughout the different steps of the value chain, precise information about the microbial populations in swine, from farm to the packaging of meat, must be obtained. To date, the only information available is limited to studies where samples were randomly or sporadically taken at specific steps of the value chain.

Several 16S rRNA amplicon sequencing studies have focused on key areas of the swine value chain. The microbial ecosystem found in the air of the housing building (Nehme et al., 2008; Kumari et al., 2016; Kraemer et al., 2019; Yan et al., 2021), the swine gut (Kim et al., 2015; Kim and Isaacson, 2015; Zhao et al., 2015; Holman et al., 2017; Crespo-Piazuelo et al., 2018; Quan et al., 2018; Xiao et al., 2018; Adhikari et al., 2019; Mark Welch et al., 2019; Vigors et al., 2020), the respiratory tract, mouth and saliva (Wang et al., 2018; Huang et al., 2019; Mou et al., 2019; Murase et al., 2019), the meat plant environment (Bridier et al., 2019; Zwirzitz et al., 2020) and the carcass (Mann et al., 2016; Zwirzitz et al., 2019; Zwirzitz et al., 2020) have been examined. Swine gut microbiota is by far the most studied of these environments, enough so that the concept of a “core” microbiota (bacteria present in over 90% of samples) was put forward by Holman et al. (2017). These authors identified the genera *Clostridium, Blautia, Lactobacillus, Prevotella, Ruminococcus, Roseburia,* RC9 “gut group” and *Subdoligranulum* as the main members of that “core”.

To our knowledge, no other study covers all of these environments and follows the same live animals from farm to meat cuts. Two commercial swine farms with different sanitary statuses were selected, we characterized the microbiota of the animals and their environments and observed how the microbiota changed at the carcass dressing (slaughter, evisceration) and meat cut preparation steps. This descriptive study presents detailed knowledge of the farm and animal microbiota, and the efficacy with which meat processing plants mitigate the impact of these microbes on the resulting carcasses and meat cuts.

## 2 Material and methods

### 2.1 Selected farms

Two commercial farms out of 126 were selected by experienced veterinarians and were located in the province of Quebec. The farms were selected based on their sanitary status, the gastrointestinal health of the animals and the medical history associated with the farms; one farm with a lower sanitary status (farm-L) and one with a higher status (farm-H). Both were finishing farms of similar housing size, with 1,200 and 1,600 animals, respectively. The animals (female (Yorkshire X Landrace) X Duroc male) were fed the same commercial diet and followed the same 4-phase finishing nutrition program. There was one exception at farm-L, where the diet changed from pellets to mash feed 22 days before the first sampling, due to an episode of salmonellosis (see discussion for clinical details). Animals from both farms were sent to the same federally-inspected meat plant when they reached a weight of 115 ± 7 kg. They came from the same production lot where three out of five shipments were followed. Samples were collected at each farm 3 days before the swine were transported to the slaughterhouse; farm-L animals were sent from July to August and farm-H from October to November. Animals were subject to a mandatory 18-h feed withdrawal before slaughter as required by the market agreement on animal welfare issues. All animal care and handling procedures were approved by Université Laval’s Animal Use and Care Committee (2019-329), which strictly adheres to the Guidelines of the Canadian Council on Animal Care (CCAC, 2009).

### 2.2 Slaughter and carcass breaking

Pigs were separated into groups of five and stunned using CO_2_. After dressing, the carcasses were blast-chilled for 90 min and then cooled overnight (24 h, 2°C) before being split into retail cuts (Supplementary Figure S1). Inspection data were collected, including frequency of demerits (abscess, lymphadenitis, bruises, etc.) and number of carasses that were condemned or retained for further examination (dead animal at reception, large and widespread abscesses, peritonitis, icterus, etc.), stomach size (in terms of feed withdrawal efficacy) and presence of bloated viscera.

### 2.3 Sampling and sample processing

Multiple types of samples were collected along the value chain (Supplementary Figure S1). All samples were stored on ice until they were processed. At the farms, sample types included air (Ar), feces (Fc), saliva (Sa), and feed (Fe). Fc and Sa samples were collected from 16 pens for all three shipments. Air was sampled using a SASS 3100 dry air sampler (Research International, Monroe, WA, United States) with a welded Standard electret filter cartridge. A volume of 10 m^3^ was collected at 300 mL/min for 33 min, three times a day (8:00, 11:00, and 14:00). The apparatus was placed on a table (1-m high) in the middle of the alley in the room where the pigs were housed. The filters were then preserved at −20°C until particle extractions were performed using the SASS 3010 particle extractor (Research International) with the recovery buffer (138 mM NaCl, 2.7 mM KCl, 0.05% Triton X-100, <0.1% NaN_3_ 10 mM Na_3_PO_4_, pH 7.4). Extractions were performed according to manufacturer instructions. In order to ensure sufficient amounts of material for subsequent DNA purification, particle solutions were pooled in equal volumes by sampling date, and then centrifuged at 4°C, 14 000 × g for 20 min (Sorvall legend XTR centrifuge, Thermo Fisher Scientific, Waltham, MA, United States). Excess supernatant was removed, and cells were stored at −80°C until DNA extraction.

Fc samples were collected using a PERFORMAbiome•GUT | PB-200 sampling kit following manufacturer instructions (DNAgenotek, Ottawa, Ontario, Canada). Freshly defecated feces from 16 random animals, one per pen, were collected every sampling day. Additional Fc samples were collected for metabolomic and short chain/volatile fatty acid analyses (see sections below). A 500-µL aliquot from each PB-200 tube was aseptically pooled according to sampling date. Individual and pooled samples were then stored at −80°C until DNA extraction. Saliva was collected using a P-151 kit (DNAgenotek) as suggested by the manufacturer. An aliquot of 250 µL from each P-151 tube was pooled by sampling date and then stored at −80°C until DNA extraction. Feed was aseptically sampled directly from eight feeders and stored in sterile 4-oz Whirlpack bags (Nasco, Madison, WI, United States). The samples were pooled to form a composite by mixing 2.5 g of feed from each feeder and then stored at −20°C until DNA extraction.

At the meat plant, pre-operational procedures were performed to ensure a clean processing line. On each processing day, the animals under study were the first to be slaughtered and their carcasses were the first to be split into retail cuts the next day. This allowed us to properly assess what contamination originated from the animals from each farm. Environmental samples were aseptically collected for each shipment before and after the animals under study were processed on the dressing line. Surface samples were obtained using a sterile sponge (Whirl-Pak^®^ Speci-Sponge^®^ Environmental Surface Sampling Bags, Nasco) that was humidified with 10 mL of sterile 2% buffered peptone water (Peptone Water, phosphate-buffered, Milipore Sigma, Oakville, Canada) and a 10 × 10 cm sterile template (3 M cattle template, USDA100, 3 M Canada, London, Malaysia). In collaboration with the quality control team, we selected sampling sites according to their HACCP plan. At evisceration, water samples were collected from a central drain under the viscera conveyer (Dev; 150 mL). As well, surface samples from a gutter post-evisceration (Gev; 300 cm^2^) and from a conveyor before the first carcass shower wash (Cev; 300 cm^2^) were obtained. Twenty-five blast chilled carcasses were sampled using surface swabs (Dc). Swabs were collected from a 100-cm^2^ surface from the hind leg near the anus, the belly and the jowl (300 cm^2^ in total), according to the Guidelines for *Escherichia coli* Testing for Process Control Verification in Cattle and Swine Slaughter Establishments (FSIS-GD-1996-0001, 2005, FDA). A second group of 25 carcasses, were sampled the following day after overnight refrigeration at 2°C (Cc). In the area where carcasses were split, water samples were collected from a central drain (Dcu; 150 mL) and surface samples at the end of the conveyor (Ccu; 300 cm^2^). During this part of the process, 25 shoulders (S) were randomly selected for sampling (Supplementary Figure S1) and a total of 450 cm^2^ was swabbed from the surface of each shoulder and from the inside section where the shoulder bone was removed.

At the laboratory, 10 mL of 2% buffered peptone water was added to each of Whirl-Pak bags containing the sponge. The content of the bag was then homogenized using a Stomacher 400C (Seward Laboratory Systems Inc., London, United Kingdom) for 2 min, at 230 rpm. An aliquot of 2 mL from each set of carcasses and shoulder samples was pooled and thoroughly mixed for microbial enumeration. Pooling was deemed necessary for some samples in order to obtain enough DNA for downstream applications. Samples were stored at −80°C until DNA extraction.

### 2.4 Short-chain fatty acid analysis of feces

Short-chain fatty acid (SCFA; acetic, propionic, butyric, isobutyric, valeric and isovaleric acids) concentrations were measured in the 16 individual Fc samples from each sampling day (96 total samples). Fc samples were stored at −80°C until analysis. Fecal suspensions were then prepared from thawed Fc samples. Samples were divided into 500 mg aliquots of feces that were dissolved in 10 times the volume of water and homogenized for 2 min with a Bead Ruptor 12 (Omni international, Kennesaw, GA, United States). The suspensions were then centrifuged at 4°C, 5,500 × g for 30 min and SCFAs were extracted from the supernatant by liquid-liquid extraction and analyzed by gas chromatography coupled to a flame ionization detector (GC-FID Shimadzu, Kyoto, Japan), as described in Roussel et al. (2022).

### 2.5 Metabolomic analysis of feces

Untargeted metabolomics was also performed on the individual Fc samples using a liquid chromatography coupled to a mass spectrometer (LC-MS). Feces were thawed and divided into 900 mg aliquots. Samples were then lyophilized with a Lyovapor L-300 (BÜCHI Labortechnik AG, Flawil, Suisse) for 72 h. A volume of 12.5 µL of 50% MeOH in water per mg of dry matter was added. Samples were then mixed with a bead beater (Bead ruptor 12) for 2 min and then agitated with a multi-tube vortex (Vx-2500, VWR international, Wayne, NJ, USA) for 5 min. Next, samples were sonicated in an ultrasonic bath at 25°C for 30 min, agitated with a multi-tube vortex for 5 min and centrifuged at 4°C, 2 795 × g for 30 min. A volume of 250 µL of supernatant was collected and mixed with 250 µL of 50% MeOH in water containing 10 internal standards (2 ppm trihydroxybenzoic acid-d_2_, 2 ppm caffeine-methyl-d_3_, 10 ppm succinic acid-d_6_, 0.2 ppm N-dodecylphosphocholine-d_38_, 10 ppm trans-cinnamic acid-d_5_, 2 ppm L-tryptophane-d_5_, 2 ppm glycocholic acid-d_4_, 10 ppm L-leucine-d_7_, 2 ppm 4-hydroxybenzoic acid-d_4_ and 2 ppm methyl 4-hydroxybenzoate-d4; CDN isotope). Finally, samples were filtered with spin filters (InnoSep Spin, NY, 0.2 µm, Canadian Life Science, Peterborough, Canada) prior to LC-MS analysis.

LC-MS analyses were performed on a system consisting of a Vanquish ultra-high-performance liquid chromatography (UHPLC) and a Fusion Tribrid mass spectrometer (Thermo Fisher Scientific, Waltham, MA, United States). Mobile phases were water (A) and acetonitrile (B), each with 0.1% formic acid. The following elution gradient was used: 0 min 2% of B, 0.5 min 2% of B, 9 min 45% of B, 9.5 min 80% of B, 15.5 min 80% of B, 16 min 2% of B and 22 min 2% of B, at a flow rate of 0.3 mL/min. A volume of 5 µL was injected on an Acquity UPLC HSS T3 Column (100 Å, 1.8 µm, 2.1 mm × 100 mm; Waters, Milford, MA, United States) and kept at 30°C. Samples were maintained at 4°C in the auto-sampler. A Quality Control (QC) pooled sample consisting of an equal volume of each sample was analyzed every 10 samples.

An electronebulizer heated to 350°C was used as an ionization source with a capillary voltage of 3.5 kV in positive mode and 2.5 kV in negative mode. Mass spectroscopy (MS) acquisitions were performed on an orbitrap at a resolution of 120 000 in profile mode using Easy-IC for mass correction. All other parameters were set at their default values. MS^2^ spectra for the QC pooled sample were acquired using the AcquireX method. Data were analyzed with the Compound Discovered 3.2 and MetFrag Web 2.1 software packages (Ruttkies et al., 2016).

### 2.6 Microbial analysis

For enumeration on agar plates, tenfold serial dilutions were carried out using sterile 0.1% peptone water (BD Biosciences, Franklin Lakes, NJ, United States). Total aerobic mesophilic (TAM) counts (Health Canada, 2020) were performed on Plate Count Agar (BD Biosciences; 35°C for 48 h). *Enterobacteriaceae* (EB) counts (Health Canada, 1997) were performed on Violet Red Bile Glucose Agar (BD Biosciences; 35°C for 24 h). Measurements were performed in duplicate. For environmental samples (drains, conveyor and gutter), contamination originating from the animals was measured by subtracting the value obtained after the pre-operation procedures from the value obtained right after the passage of animals under study. The number of animals sent to the meat plant varied over the six shipments, therefore basic statistical weighting was used to adjust the results to a lot size of 300 animals (Kalton and Flores-Cervantes, 2003). All bacterial counts were transformed to a Log_10_ value of colony forming units per 300 cm^2^ or 300 mL prior to statistical analysis (Gill, 2000). When no colonies were observed, the level of detection was used in statistical analyses.

### 2.7 DNA extraction and 16S rDNA sequencing

Frozen samples were thawed at 4°C. Liquid samples were centrifuged (Sorvall legend) at 4°C, 24 000 × g for 10 min for the 50 mL samples, and 14 000 × g for 20 min for the 10 mL samples. The supernatant was removed, and the cell pellets were used for DNA extraction. Fe samples were hydrated in 2% buffered peptone water in a 9:1 ratio of water to feed for 30 min at 4°C in filtered stomacher bags. The hydrated pellets were homogenized using a Stomacher 400C for 2 min at 230 rpm. A total volume of 2.5 mL was recovered and centrifuged at 14 000 × g for 10 min at room temperature. The pelleted cells were used for DNA extraction.

DNA extraction kits were chosen based on their extraction efficiency with the various samples collected. A DNeasy PowerSoil Kit (QIAGEN, Toronto, Canada) was used for air, feed, conveyor, gutter, and carcass samples. A QIAamp Fast DNA Stool Mini Kit (QIAGEN) was used for the stabilized feces, the DNeasy PowerWater Kit (QIAGEN) for the liquid collected from the drains and a QIAamp BiOstic Bacteremia DNA Kit (QIAGEN) was used for S samples. A MasterPure Complete DNA and RNA Purification Kit (Lucigen, Teddington, United Kingdom) was used for Sa samples. All kits were used following the manufacturer protocols. Extracted DNA samples were quantified using a NanoDrop One spectrophotometer (Thermo Fisher Scientific, Ottawa, ON, Canada).

### 2.8 Amplification of the 16S DNA

During the preliminary set up of the experiment, metagenomic shotgun sequencing was performed to analyze the microbial communities. Unfortunately, pig DNA was in such abundance (>95%) in the samples collected that the analysis was losing depth. Therefore, amplification and sequencing of the 16S gene was deemed more appropriate. Library preparation and sequencing were performed at the *Plateforme d’analyse génomique* (*Institut de Biologie Intégrative et des Systèmes*, Université Laval, Quebec City, Canada). Amplification of the 16S V3-V4 region was performed as described in Klindworth et al. (2012) in a long oligo PCR approach. The PCR reactions were purified using an Axygen PCR cleanup kit (Axygen, New York, NY, USA). The quality of the purified PCR product was confirmed with a DNA7500 BioAnalyzer chip (Agilent, Santa Clara, CA, United States) and quantified using a NanoDrop One spectrophotometer. Barcoded amplicons were pooled in equimolar concentrations and sequenced on an Illumina MiSeq (paired-end 300 bp with two index reads). Samples collected after pre-operational cleaning and disinfection procedures with insufficient read quantity were discarded.

### 2.9 Sequence processing

The first step in read processing was to inspect the quality plots generated by FastQC (version 0.11.5; Andrews, 2010). Amplicon sequence variants (ASVs) were generated using the DADA2 workflow package (version 1.22.0; R version 4.1.1; Callahan et al., 2016). During filtration, the first 17 nucleotides of the forward reads and the first 21 of the reverse reads were trimmed to remove primers. Sequences containing ambiguous nucleotides (N) were discarded. According to the findings of Prodan et al. (2020), the expected error filter was not used to avoid bias towards bacteria with an error-prone genome. Dereplication, sample inference, chimera identification and merging of the paired-end reads were performed using the default parameters, with the exception that samples were pooled during inference step. Taxonomic assignment was done using the SILVA rRNA database (release 138.1; Pruesse et al., 2007) with the naive Bayesian classifier method (the assignTaxonomy command of the DADA2 package). Species were then added with the add species function. A phylogenetic tree was built based on a multiple alignment (DECIPHER R package version 2.22.0; Wright, 2016). Then a neighbor-joining tree was built and used as a basis for the GTR + G + I maximum likelihood tree (phangorn, R package version 2.8.1; Schliep, 2010). Counts, taxa, study metadata and phylogenectic trees were then combined into a phyloseq object (version 1.38.0; McMurdie and Holmes, 2013). Contaminants were removed using blank samples and the prevalence method (threshold of 0.5; decontam, R package version 1.14.0; Davis et al., 2018). Data were filtered by removing non-bacterial ASVs (Kingdom Eukaryota and Archaea, Order Chloroplast and Family Mitochondria), then ASVs without phylum identification followed by any low prevalence phylum (less than five ASVs per phylum), and finally low prevalence ASVs (present in less than 5% of samples). Filtered ASVs were searched against the NCBI nr/nt 16S curated database (Bioproject 33175 or 33317; excluding archea; downloaded 14-01-2022) in GenBank using BLASTN (version 2.12.0; Altschul et al., 1990). When the query ASV had more than 97% identity with the sequences in the GenBank database, the same genus level identification as in the SILVA database and a clearly defined species assignment (no ambiguous same percentage identity yet different species identification; those were left as NA) was manually reassigned to that ASV. The same procedure was followed for genus reassignment with a 90% identity threshold and family consensus. The remaining taxa were grouped in the “remaining taxa” row. The ASV counts were normalized into relative abundance for heatmap visualisation (ampvis2, R package version 2.7.13; Andersen et al., 2018). A heatmap graph was produced using a subset of bacterial families known to impact meat (*Campylobacteraceae*, *Carnobacteriaceae, Enterobacteriaceae, Lactobacillaceae* and *Staphylococcaceae;* Baer et al., 2013; Saucier, 2016; Møretrø and Langsrud, 2017). Sequences can be found in DDBJ/ENA/GenBank under the BioProject PRJNA923296.

### 2.10 Statistical analysis

A Shapiro-Wilk test was used to confirm normality and a Student T test to compare each sample type between farms and for both TAM and *Enterobacteriaceae* counts. T tests were also used for samples collected before and immediately after the animals under study were processed as well as carcass inspection data collected for each shipment and SCFAs in feces. These tests were also performed without separating them by farm in order to determine whether the animal’s presence had an effect on the contamination level. A non-parametric analysis was also performed on the basis that when a specific microbial concentration threshold is used to determine the end of shelf life, cell counts closer to that threshold limit represent a greater microbial food safety risk. For that, a Wilcoxon test was performed to compare the two farms instead of a Student T test because the distributions were not normal when all sample type were pooled together.

Metabolomics data integrity was first validated by measuring the %CV on different internal standards detected in positive (caffeine-d_3_, dodecylphosphochloline-d_38_, tryptophan-d_5_ and leucine-d_7_) and negative (succinic acid-d_6_, trans-cinnamic acid-d_5_ and tryptophan-d_5_) ionization. The %CV was below 20% for all these compounds. Then, untargeted metabolomic data quality was assessed using a principal component analysis (PCA) and showed a strong clustering of QC-pool injections. One sample was considered an outlier and was removed. Metabolic features were filtered based on the following strict criteria: present in all the QC pool injections (3183 and 542 features left in positive and negative mode), and %CV < 20% in its own group (farm-L or farm-H, 251 and 71 features left in each ionization mode). Features without a MS^2^ spectra were also removed from the analysis (240 and 64 features left). A differential analysis (Log_2_ fold change >1 and a *p*-value <0.05) was performed on the remaining features. Selected metabolites were then putatively identified using the Compound Discoverer FiSH score as the scoring metric and then confirmed using MetFrag Web.

The 16S rDNA sequences were categorized based on farm, sample type (Ar, Fc, Sa, Fe, Dev, Cev, Gev, Dc, Cc, Dcu, Ccu, and S), sampling location (farm, evisceration, cut-out) and shipment week (weeks 1, 2, and 3). Differential abundance analyses were performed also for a subset of genera and families important for meat safety (*Campylobacteraceae*, *Carnobacteriaceae, Enterobacteriaceae, Lactobacillaceae*, and *Staphylococcaceae),* between farms, using the Analysis of Compositions of Microbiomes with Bias Correction (ANCOMBC) methodology. This method was performed on the raw read counts of each family and their genus (ANCOMBC R package version 1.4.0; Lin and Peddada, 2020). Several parameters were used to control the false discovery rate (FDR) and increase the robusnes of the analyses. These included: a zero cut of 1 (no genera excluded), 1,000 iterations, a conservative variance estimate, FDR adjustment of *p* values, structural zeros and negative lower-bound zeros.

Alpha diversity (within-sample) was calculated on non-normalized data (phyloseq version 1.30.0; McMurdie and Holmes, 2013). The species richness was evaluated with an Observed and Chao1 index and evenness was evaluated with the Shannon and Simpson index. Theses indices were selected to evaluate large population changes as they give both a number for the total ASV (Observed and Chao1) and abundance distribution amongst these ASV (Shannon and Simpson). A Student T test was used to compare each sample type between farms. A FDR correction was used to control false positives. To evaluate differences between sample type diversity, a Tukey HSD test was performed between all the samples (agricolae, R package version 1.3–5; Mendiburu and Yaseen, 2021).

Beta diversity (between samples) was calculated for the normalized ASV counts using unweighted and weighted UniFrac distances (Lozupone and Knight, 2005) and Bray-Curtis dissimilarities (Phyloseq R package version 1.38.0; McMurdie and Holmes, 2013). Data were normalized by performing a Hellinger transformation (decostand function of the vegan R package version 2.5–7; Legendre and Gallagher, 2001). Principal coordinate analysis (PCoA) was used to visualize the distances between samples (Ampvis2, R package version 2.7.13; Andersen et al., 2018). Permutational analysis of multivariate dispersions (PERMDISP) was used to test the homogeneity of dispersion for each metadata category (betadisper function of the vegan R package). Since heterogeneity of dispersion was confirmed, analysis of similarities (ANOSIM; function of the vegan R package) was performed.

The linear discriminant analysis effect size (LEfSe) method was performed on non-normalized data (raw ASV counts) using the microbiome analyst platform (Dhariwal et al., 2017). Genera with higher relative abundances in the different sampling sites of the value chain were identified by LEfSe. The size effect of each of these genera was calculated using linear discriminant analysis (LDA; Segata et al., 2011). An LDA score (Log_10_) of 1.0 was used as the cut-off for identifying biomarkers. LEfSe was also used to compare the Fc samples between farms in order to identify bacteria that could be linked to short-chain fatty acid production. Here, a LDA of 2 was used as the cut-off value.

Microbial source tracking was achieved for Dc, Cc and S samples using the SourceTracker software package (version 1.0.1) with the default parameters (Knights et al., 2011). A rarefaction value of 1,000 reads and an alpha 1 and 2 of 0.001 were used. Farm (Ar, Fc, Sa, Fe) and environment (Dev, Cev, Gev, Dcu, Ccu) samples were considered source samples and those from meat (Dc, Cc, S) were considered to be sink samples.

## 3 Results

### 3.1 Characterization of the sampled farms

**FIGURE 1:**
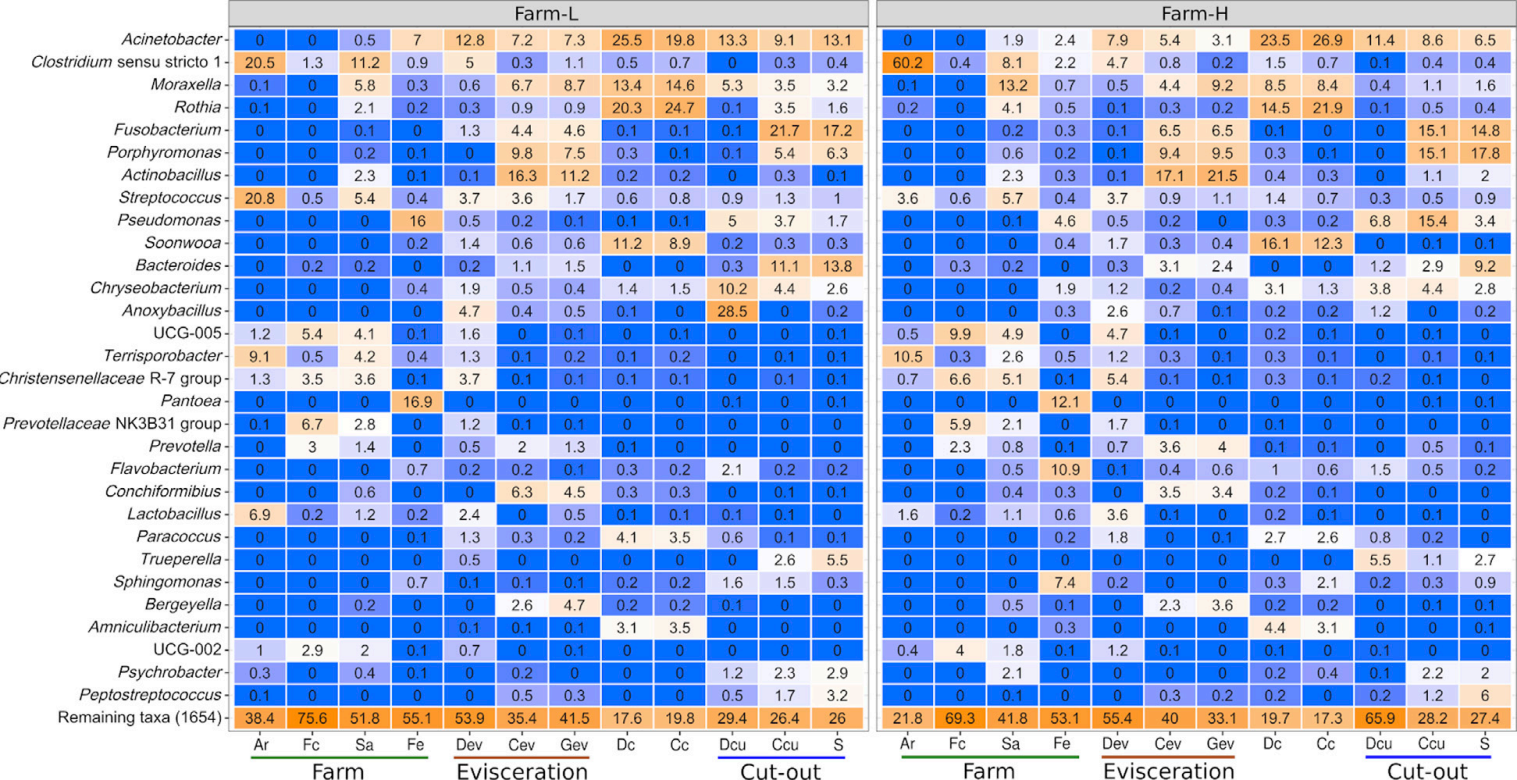
Top 30 genera based on relative abundance (%) identified in each of the sample types for both farms. Genera were identified using the SILVA database. Colour gradients range from blue = 0% to orange = 100%. Samples were collected from air (Ar), feces (Fc), saliva (Sa), feed (Fe), drain at evisceration (Dev), conveyor at evisceration (Cev), blood collection gutter (Gev), dressed carcasses (Dc), cold carcasses (Cc), drain at cut-out (Dcu), conveyor at cut-out (Ccu) and shoulder (S). Farm-L with a lower and farm-H with a higher sanitary status, respectively.

Farm microbiota were characterized by analyzing Ar, Fc, Sa, and Fe samples using 16S rRNA gene amplicons. At the genera level, samples from Ar were similar to Fc, while Sa samples were similar to Fe (Figure 1). When comparing the two farms for each sample type, the Fe microbiota, and to a lesser extent Sa, exhibited noticeable differences. Interestingly, at the phyla level, *Proteobacteria* represented 90.7% of the reads in Fe from farm-L and 57.6% from farm-H. *Proteobacteria* were two times more abundant in Sa samples from farm-H compared to farm-L (Supplementary Figure S2).

Short-chain fatty acid concentrations in the feces of animals from both farms were measured and compared to assess microbial activity (Table 1). Concentration of total SCFAs was significantly higher in samples from farm-L than farm-H (*p* = 0.02) as well as for each individual SCFAs (all *p* < 0.01). Furthermore, the results from the LEfSe analysis indicate a higher abundance (*p* < 0.05, LDA score cutoff of 2; data not shown) of specific genera known to produce SCFAs in farm-L *Selenomonas* (Wang et al., 2012), *Anaerovibrio* (Holman et al., 2021)*, Roseburia* (Puertollano et al., 2014; Wang et al., 2019; Markowiak-Kopeć and Śliżewska, 2020), *Akkermansia* (Li et al., 2021) and *Clostridium* (Puertollano et al., 2014; Wang et al., 2019; Markowiak-Kopeć and Śliżewska, 2020).

**TABLE 1:** Average short-chain fatty acid concentrations (SCFAs) in the feces of swine from two farms with different sanitary statuses.

	Farm-L	Farm-H	SEM^a^	*p*-value^b^
Total SCFAs (mMol/kg)	128	115	3.9	0.02
SCFA (mol/100 mol)				
Acetate	49.10	56.80	0.73	<0.0001
Propionate	24.10	22.50	0.26	<0.0001
Butyrate	18.50	11.70	0.82	<0.0001
Isovalerate	3.33	3.92	0.12	0.0002
Valerate	2.83	2.57	0.01	0.01
Isobutyrate	2.12	2.51	0.08	<0.0001

^a^ SEM, standard error of the means.

^b^ *p*-value calculated using a Student T test is deemed significant at a value of 0.05.

**FIGURE 2:**
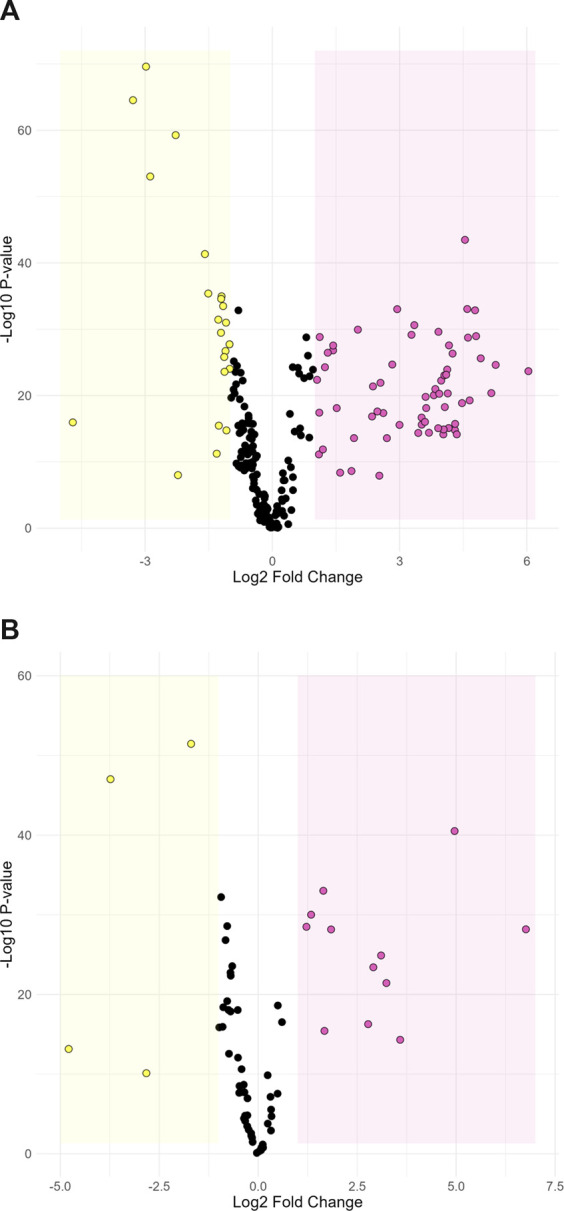
Volcano plot comparing metabolite abundance between farm-L and farm-H under positive **(A)** and negative **(B)** ionization. Dots in the yellow section are ions that are significantly more abundant in farm-H samples and dots in the pink section are significantly more abundant in farm-L samples. The highlighted sections represent a Log_2_-fold change greater than 1 and a *p*-value less than 0.05.

Metabolomic analysis was performed on feces to identify potential metabolic markers related to the farm of origin and their different sanitary statuses. There was a clear separation of data from the two farms on the first principal component of the PCA score plot for both positive (27.1%) and negative ionization (28.5%; Supplementary Figure S3). This indicates that the farm environment and characteristics had an important influence on the metabolite composition of feces. Discriminant analysis through Volcano plots provide another clear indication of the different metabolites characterizing each farm. Under positive ionization, 85 ions were significantly more abundant in the feces collected from either farm (Figure 2A). Under negative ionization, 16 ions were significantly more abundant (Figure 2B). Putative identification based on molecular mass and fragmentation spectra was obtained for 21 metabolites in positive ionization and six metabolites in negative ionization (Supplementary Table S1). Since the farms fed animals different diet form (pellet vs. mash) prior to slaughter and only two farms were sampled, we could not confidently ascribe specific biomarkers to the health status of the animals. Nonetheless, they represent potential candidates for further studies. Overall, our results indicate that the microbiota of animals were different between the two farms.

### 3.2 Characterization of the meat plant prior to operations

At the beginning of each slaughter and carcass breaking day, before the animals enter the processing line, most of the samples from the meat plant environment (Cev, Gev, Dcu, Ccu) had microbial counts (TAM and EB) around or below the detection level (<2.48 Log_10_ CFU/300 mL or cm^2^). This was true for all sampling weeks for both farms. Furthermore, there was no significant difference (*t*-test and Wilcoxon; *p* > 0.05) in the initial contamination of the meat plant on days that animals from either farm were processed. For many of the samples, DNA could not be extracted in sufficient quantity to be analyzed due to low levels of contamination. A sampling area much larger than 300 cm^2^ would have been necessary to obtain enough DNA. Despite the similarities described above, Dev samples had TAM count means of 5.17 ± 1.58 and 2.96 ± 0.24 Log_10_ CFU/300 mL and EB means of 3.93 ± 1.35 and 2.88 ± 0.70 Log_10_ CFU/300 mL for farm-L and farm-H, respectively. Initial counts for Dev appear to vary substantially between sampling days after the pre-operation procedures.

### 3.3 Microbial contamination from presence of animals on the processing line

Unsurprisingly, as the animals went through the processing line, the CFU in environmental samples rose signficantly. For TAM counts, the increase was significant (*p* < 0.05) for every sample. The EB counts in the Gev (*p* = 0.08) and the drain at cut-out (Dcu;*p* = 0.4) samples were not significantly different from those collected from the clean processing line.

**FIGURE 3:**
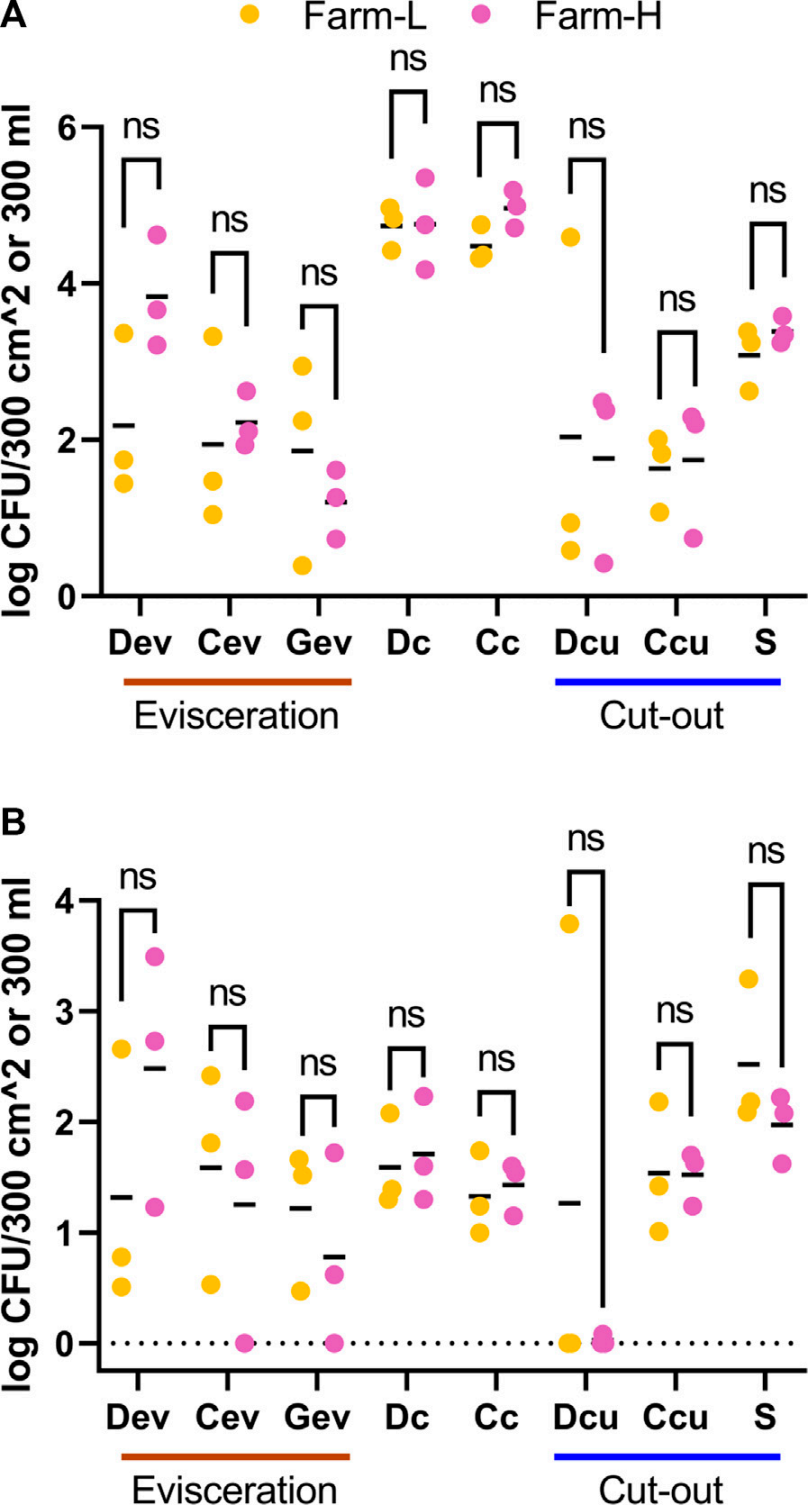
Total aerobic mesophilic bacteria **(A)** and *Enterobacteriaceae* **(B)** counts in Log_10_ CFU/300 cm^2^ or 300 mL for samples associated with farm-L (yellow) or farm-H (pink), with lower and higher sanitary statuses, respectively. Samples were collected from air (Ar), feces (Fc), saliva (Sa), feed (Fe), drain at evisceration (Dev), conveyor at evisceration (Cev), blood collection gutter (Gev), dressed carcasses (Dc), cold carcasses (Cc), drain at cut-out (Dcu), conveyor at cut-out (Ccu) and shoulder (S) after the last animal under study was processed. The results are the contamination from animals, calculated by subtracting the values obtained for the clean production line. The detection level for drain samples was ≤2.48 Log_10_ CFU/300 mL for TAM and EB. For the other samples, the detection level was ≤1.22 Log_10_ CFU/300 cm^2^ for TAM and ≤0.70 Log_10_ CFU/300 cm^2^ for EB.

To evaluate contamination of the processing line due to animals, the microbial counts from environmental samples collected after animals under study were processed were subtracted from those measured at the end of the pre-operation procedures. No significant difference (*t*-test and Wilcoxon) was detected between the farms for any of the microbial counts (Figure 3). There was only a marginal tendency for the cold carcass TAM counts (*p* = 0.067; *t*-test), where farm-L had a mean of 4.48 ± 0.24 Log_10_ CFU/300 cm^2^ and farm-H had a mean of 4.96 ± 0.24 Log_10_ CFU/300 cm^2^, but the difference was below 1 Log_10_ unit. For Dc, Cc and S samples, TAM counts diminished as the samples were collected further along the processing lines. Means decreased from 4.74 (Dc) and 4.48 (Cc) to 3.08 (S) Log_10_ CFU/300 cm^2^ for farm-L, and 4.76 (Dc) and 4.96 (Cc) to 3.39 (S) Log_10_ CFU/300 cm^2^ for farm-H. However, EB counts did not follow this trend. Samples increased from a mean of 1.59 (Dc) and 1.33 (Cc) to 2.52 (S) Log_10_ CFU/300 cm^2^ for farm-L and 1.71 (Dc) and 1.43 (Cc) to 1.97 (S) Log_10_ CFU/300 cm^2^ for farm-H.

Various non-microbiological data were collected by inspectors to assess carcass weight and other potential defects (see above for description; data not shown). No significant differences were observed between the two farms for any of the inspection data collected.

### 3.4 Analysis of microbiota variations across the swine value chain using 16S rDNA amplicons

Overall, *Acinetobacter* was the most abundant genus across all samples except for in Ar and Fc (Figure 1). These bacteria were found mostly on the surface of dressed (Dc) and cold carcasses (Cc; between 20% and 27% of relative abundance) and were detected at lower abundances across all meat plant environmental samples. *Clostridium* (*Clostiridum*_sensu_stricto_1) was particularly abundant in Ar samples (20.5% and 60.2% for farm-L and H, respectively). *Fusobacterium*, *Porphyromonas* and *Bacteroides* were mainly detected on equipment surfaces (Cev, Gev, and Ccu) and S samples. Results varied for Ccu and S samples depending on the farm. *Streptococcus* was the only genus found across the whole value chain. This genus generally had low abundances (<5.7%) except for farm-L air samples (20.8%). *Pseudomonas* distribution varied greatly between samples. In Fe samples, *Pseudomonas* was more abundant at farm-L (16.0%) than farm-H (4.6%). The opposite was found for Ccu (3.7% at farm-L, 15.4% at farm-H). *Anoxybacillus* abundance was different between the two farms for Dcu (28.5% at farm-L and 1.2% at farm-H). The *Anoxybacillus* high mean relative abundance at farm-L was caused by one sample with extremely high abundance. This result was consistent with the abnormally high plate counts for TAM and EB in the same contaminated sample (Figure 3; Dcu).

**FIGURE 4:**
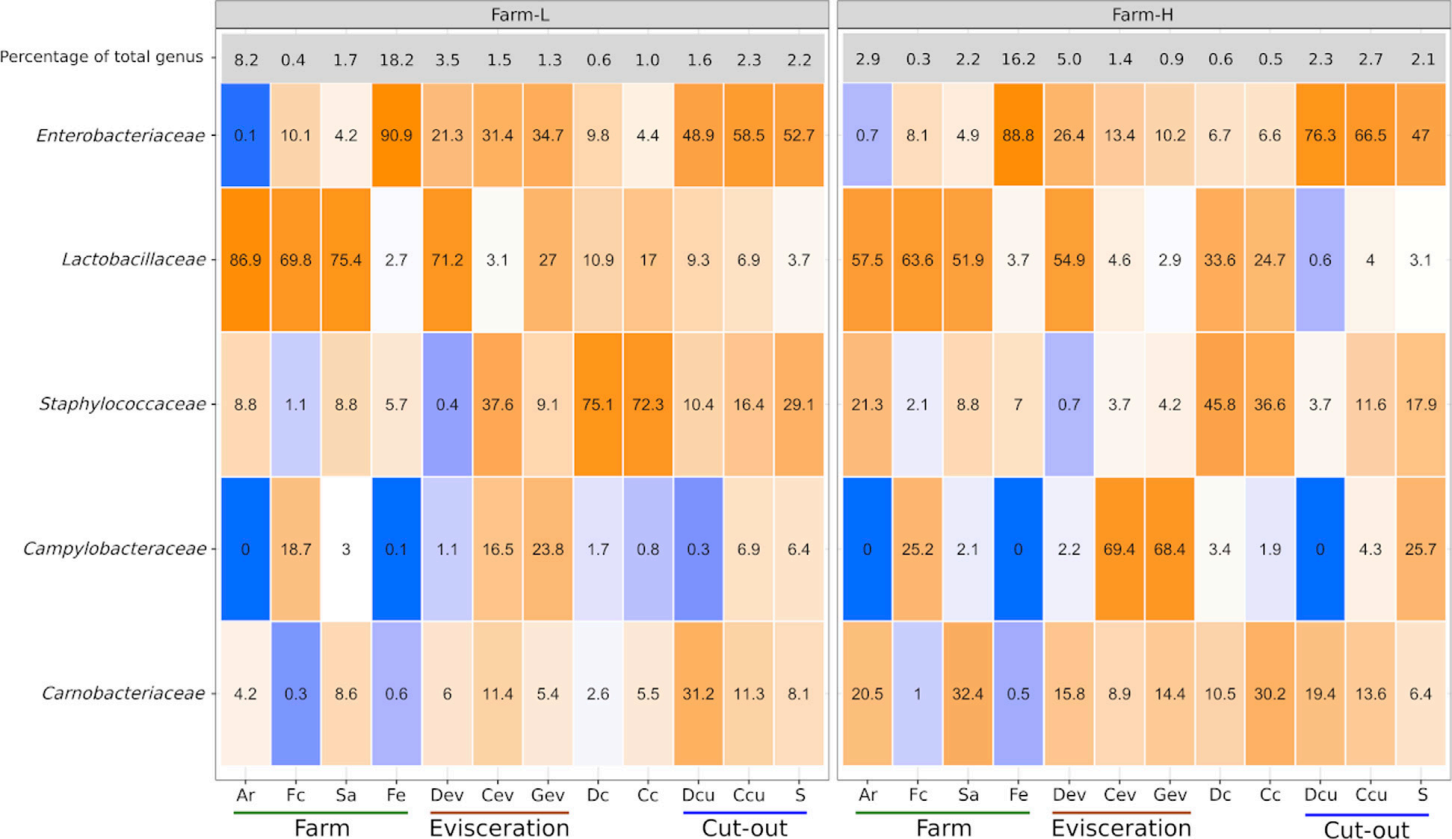
*Campylobacteraceae*, *Carnobacteriaceae*, *Enterobacteriaceae*, *Lactobacillaceae*, and *Staphylococcaceae* relative abundances (%) to one another calculated for each of the sample types for both farms. The top row indicates the percentage that these five families represented in the total microbiota of each sample type. Colour gradients range from blue = 0% to orange = 100%. Samples were collected from air (Ar), feces (Fc), saliva (Sa), feed (Fe), drain at evisceration (Dev), conveyor at evisceration (Cev), blood collection gutter (Gev), dressed carcasses (Dc), cold carcasses (Cc), drain at cut-out (Dcu), conveyor at cut-out (Ccu) and shoulder (S). Farm-L with a lower and farm-H with a higher sanitary status, respectively.

Certain bacterial families have a large impact on the pork value chain from a meat safety and hygiene perspective. Some cause meat spoilage while others increase meat shelf life. Five of those families, *Campylobacteraceae, Carnobacteriaceae, Enterobacteriaceae*, *Lactobacillaceae* and *Staphylococcaceae,* were examined as a subset of the total data. The relative abundances of the genera within this subsample are presented in Figure 4. No *Listeriaceae* were detected. The abundances of these families were similar between the two farms. *Campylobacteraceae* were found mostly in Fc, Cev, Gev, and S samples. *Carnobacteriaceae* were mostly detected at the cut-out area, but were also found across the value chain. Only traces were found in Fc and Fe. *Enterobacteriaceae* were present only in trace amounts in Ar samples. High abundances of *Lactobacillaceae* were identified in Ar, Fc, Sa, Dev and on carcass surfaces. *Staphylococcaceae* were found across the value chain, but only in trace amounts in the Fc and Dev. At the genus level, *Allicoccus*, *Atlantibacter*, *Companilactobacillus*, *Corticicoccus*, *Cronobacter*, *Mamallicoccus*, *Serratia*, *Salinicoccus*, *Salmonella*, and *Weisella* (*p* < 0.001) were significantly more abundant in farm-L samples. The only genus that was more abundant in the farm-H samples was *Lacticigenium* (*p* < 0.001; Supplementary Figure S4). *Escherichia/Shigella* were detected for both farms mainly in Sa and Fc, and in Dev, Cev and Gev. The same *Escherichia/Shigella* ASV detected in Sa and Fc was also found in the meat plant environment (ASV168; Supplementary Table S2). Contrary to *Campylobacter*, only traces of *Escherichia/Shigella* were detected on carcasses and the resulting shoulder meat cuts.

**FIGURE 5:**
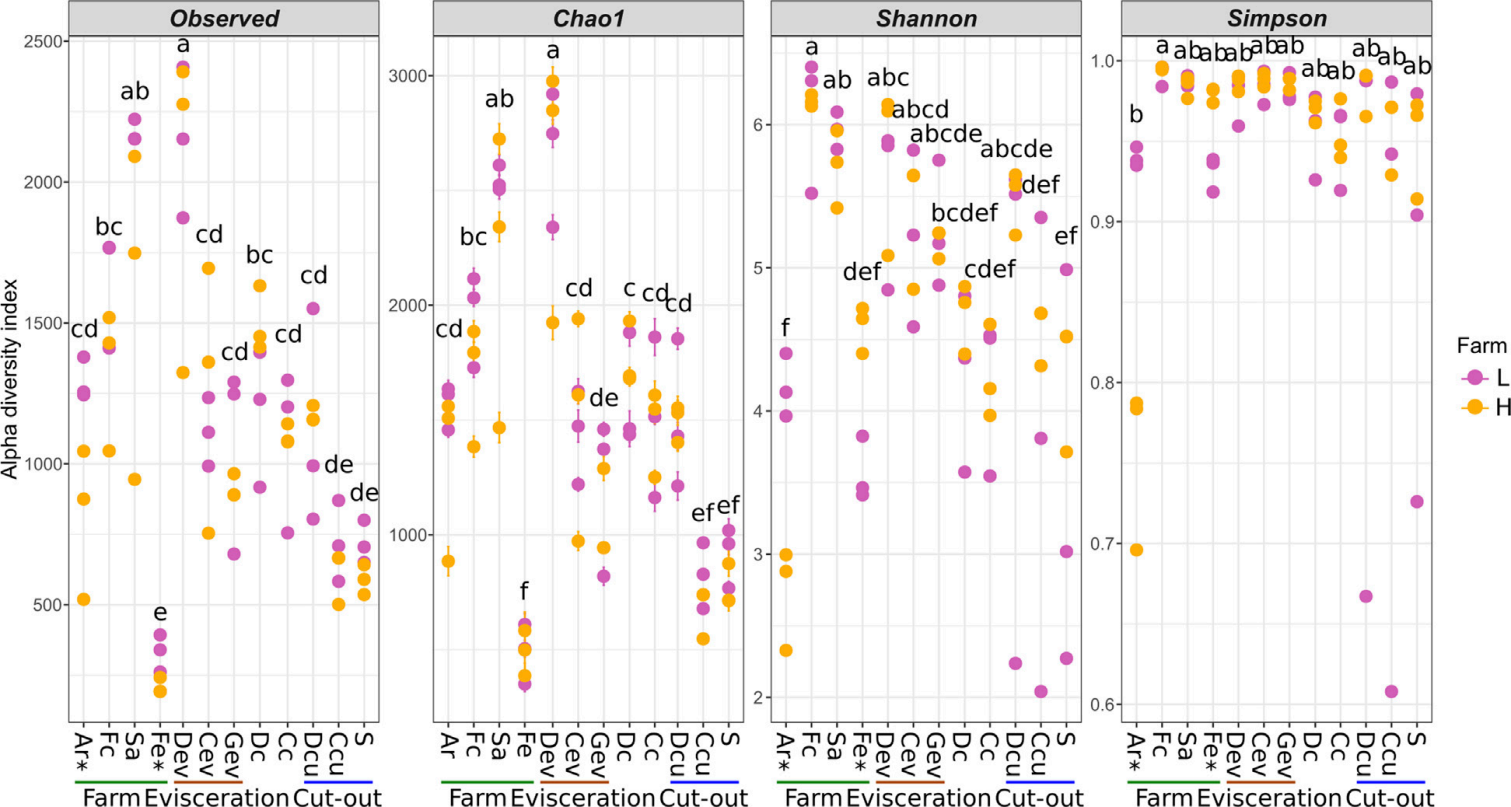
Alpha diversity of the samples along the value chain calculated with different indexes for richness (Observed, Chao1) and both richness and evenness (Shannon, Simpson). Significant differences between the two farms according to a Student T test (*p* < 0.05) are identified with an asterisk (*) at the bottom of the graph. Significantly different samples are identified with different letters according to a Tukey HSD test (*p* < 0.05). Samples were collected from air (Ar), feces (Fc), saliva (Sa), feed (Fe), drain at evisceration (Dev), conveyor at evisceration (Cev), blood collection gutter (Gev), dressed carcasses (Dc), cold carcasses (Cc), drain at cut-out (Dcu), conveyor at cut-out (Ccu) and shoulder (S). Farm-L (yellow) or farm-H (pink) with lower and higher sanitary statuses, respectively.

Alpha diversity was determined for each sample type to evaluate microbial diversity in terms of richness (Observed and Chao1) and both richness and evenness (Shannon and Simpson; Figure 5). There were significant differences between farms for Ar and Fe samples (*p* < 0.05). Diversity was lowest in Fe samples and highest in Sa samples (*p* < 0.05; Observed and Chao1). In terms of evenness, the microbiota in the Ar was more uniform for farm-L than for farm-H, while the reverse was observed for the Fe samples. The Simpson index for the Ar samples was distinctive from the other sample types and indicated a clear prevalence of a few taxa, namely, *Clostridium* in farm-H and *Clostridium* and *Streptococcus* in farm-L (Figure 1). As samples were obtained along the different locations and steps in the value chain, there was a clear reduction in microbial diversity. However, there was a large variation in the Shannon and Simpson indexes for farm-L suggesting that the microbiota varied substantially between the three shipment weeks.

**FIGURE 6:**
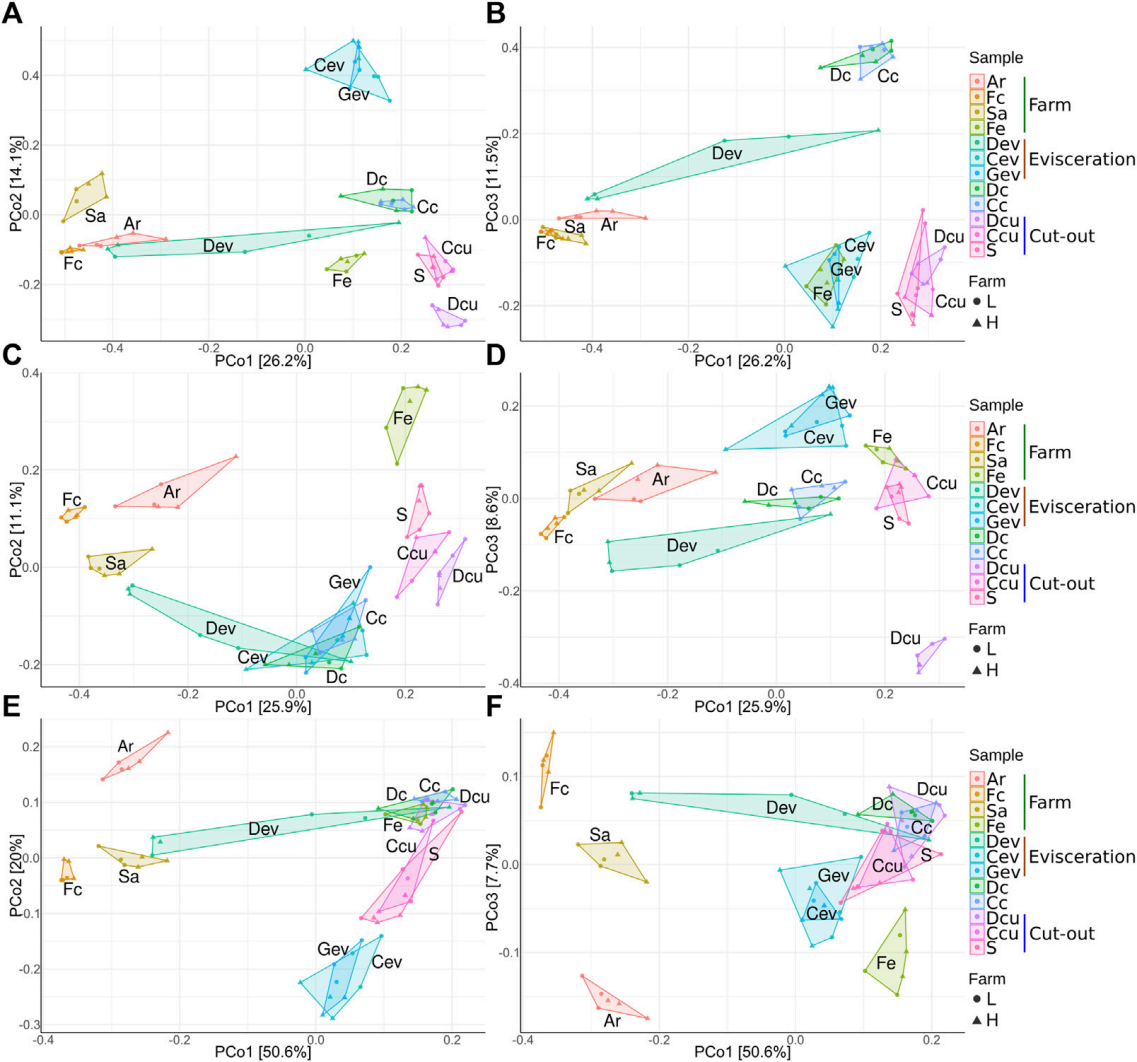
Principal-coordinate analysis plots of Bray-Curtis dissimilarities **(A,B)**, unweighted UniFrac distances **(C,D)**, and weighted UniFrac distances **(E,F)** classified by sample type. The right side **(A,C,E)** are axes 1 and 2 and the left side **(B,D,F)** are axes 1 and 3. Samples were collected from air (Ar), feces (Fc), saliva (Sa), feed (Fe), drain at evisceration (Dev), conveyor at evisceration (Cev), blood collection gutter (Gev), dressed carcasses (Dc), cold carcasses (Cc), drain at cut-out (Dcu), conveyor at cut-out (Ccu) and shoulder (S). Farm-L or farm-H with lower and higher sanitary statuses, respectively.

Beta diversity analysis was conducted to visualize the between-sample differences in diversity and to identify which factors impact the changes in the microbiota along the value chain (Table 2). Unweighted and weighted UniFrac distances and Bray-Curtis dissimilarities were visualized using PCoA (Figure 6) and an ANOSIM test (*p* < 0.05) on the distance matrices. Across all distance measures, sample type and location (farms, evisceration area and cut-out area samples) were significant (*p* < 0.0001) while farms (L and H) and shipment weeks (1, 2, and 3) were not. Sample type had the highest R value indicating that it explained the largest amount of variation. Each sample type had a unique microbiota. They then regrouped themselves based on their position along the value chain (Figure 6). This secondary grouping is defined by the unique microbiota at each farm, the evisceration area, and the cut-out area. In all distance metrics, samples were mostly distributed along the principal coordinate 1 (PCo1) according to their location, indicating that the microbiota at the farms are replaced and became less diverse further down the value chain. The only exceptions were Dev and Fe samples. The Dev samples varied between farms and were distant from the other samples from the meat plant. The Fe samples were more similar to samples from the meat plant than other samples from the farms. Principal coordinates 2 (PCo2) and 3 (PCo3) appear mostly linked to dissimilarities between the different sample types. When considering only abundance (Bray-Curtis dissimilarities; Figures 6A, B), the PCo2 microbiota were mostly influenced by the dissimilarities between Cev and Gev, which is maximal when compared to Dcu. For PCo3, dissimilarities between carcass samples (Dc and Cc) and the rest of the meat plant samples were apparent. When only considering phylogeny (unweighted UniFrac distances; Figures 6C, D) along the PCo2, samples were distributed mostly based on the dissimilarity between Fe and the meat plant samples. On the PCo3 axis, dissimilarities between the Dcu and the rest of the samples were evident. When both abundance and phylogeny is considered (weighted UniFrac distances; Figures 6E, F), the samples were distributed mostly along the PCo2 according to their similarities to Ar or Cev and Gev samples. Along the PCo3, smaller variations are illustrated (axis is narrower) through minor differences between Fe and Ar samples.

**TABLE 2:** Factors associated with the microbiota community structure for samples collected along the swine value chain as measured using ANOSIM of the weighted and unweighted UniFrac distances and Bray-Curtis dissimilarities (R value).

Parameters	Weighted UniFrac		Unweighted UniFrac		Bray-curtis	
	R value	*p*-value^a^	R value	*p*-value	R value	*p*-value
Sample types^b^	0.871	0.0001	0.859	0.0001	0.896	0.0001
Locations^c^	0.449	0.0001	0.494	0.0001	0.589	0.0001
Farms	−0.012	0.6998	0.015	0.1653	0.015	0.1717
Shipment weeks^d^	−0.037	0.9999	−0.031	0.9948	−0.035	0.9978

^a^ *p*-value is significant at a level of 0.05.

^b^ Sample types (12): air, feces, saliva, feed, drain at evisceration, conveyor at evisceration, blood collection gutter, dressed carcasses, cold carcass, drain at cut-out, conveyor at cut-out and shoulder.

^c^ Locations (3): farms, evisceration area and cut-out area samples.

^d^ Shipment weeks 1, 2, and 3.

**FIGURE 7:**
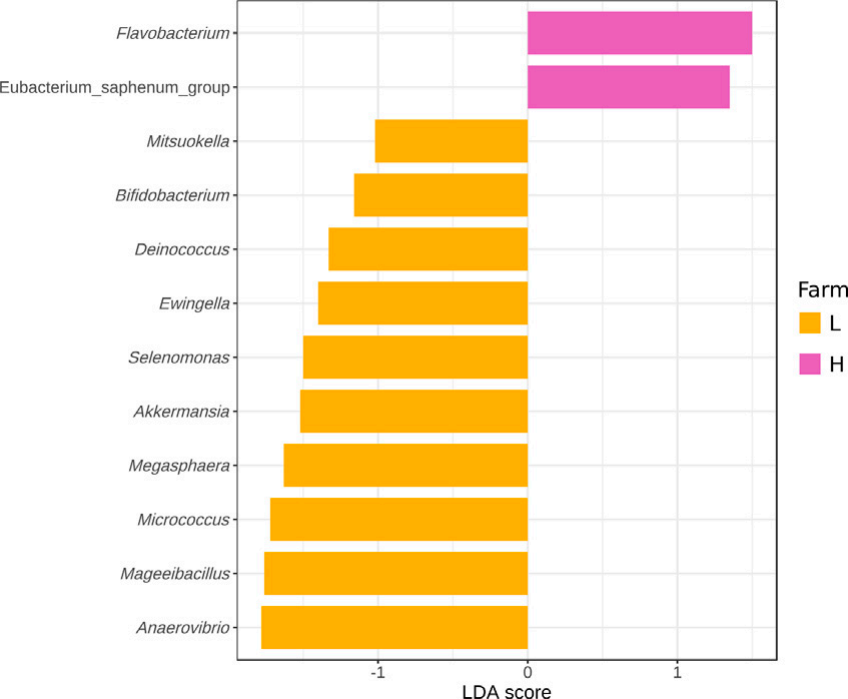
Differentially abundant genera across all samples as assessed using linear discriminant analysis (LDA) with effect size (LEfSe) measurements for farm-L (yellow) and farm-H (pink) with lower and higher sanitary statuses, respectively. Only those genera with an LDA score (log_10_) of >1.0 are displayed.

LEfSe analysis was performed in an effort to identify genera that could be used as biomarkers of the farm of origin. No genera were strongly associated with either farm using standard LEfSe parameters (*p* < 0.05 for the Kruskal-Wallis test and LDA score cut-off of 2). When the severity of the LDA score cutoff was lowered to 1, 12 genera were associated with a specific farm (Figure 7). *Flavobacterium* and *Eubacterium_saphenum*_group were associated with farm-H. *Anaevibrio, Mageeibacillus, Micrococcus, Megasphaera, Akkermansia, Selenomonas, Ewingella, Deinococcus, Bifidobacterium* and *Mistsuokella* were associated with farm-L. The genera associated with farm-L were found across the entire value chain, whereas the biomarkers for farm-H were mostly present in the meat plant samples. These genera represented a small percentage (<4.5%) of the total relative abundance in all sample types. The one exception is the Fe samples where they make up a larger percentage (8.3% at farm-L and 10.9% at farm-H; Supplementary Figure S5). Of the genera identified, only *Anaevibrio, Mageeibacillus, Micrococcus, Megasphaera, Akkermansia* and *Flavobacterium* were over the threshold of 1.5 LDA score. None of the genera identified here had an LDA score of 2 or higher. This indicates that while the Kruskal-Wallis test lists these bacteria as significantly more abundant at one of the farms, linear discriminant analysis shows that they had low relevancy as biomarkers of the farm origin. Inversely, this means that the bacteria that impact the value chain were not affected by which farm they were associated with. This finding is consistent with the results from the ANOSIM analysis (Table 2). However, since only two farms were processed in one plant, that does not mean it is possible, for all commercial organizations, to produce similar meat microbial quality products from animals coming from two different farms.

**FIGURE 8:**
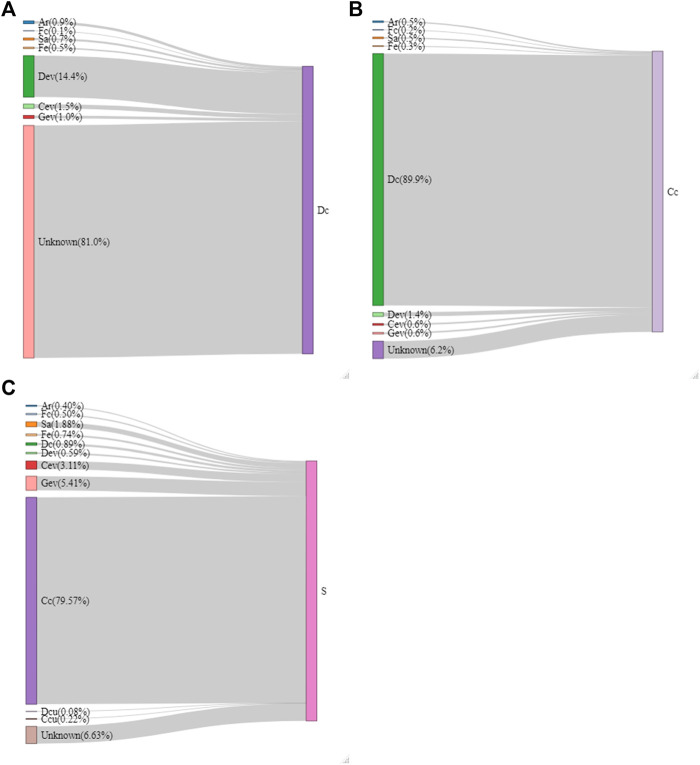
Flow diagram of the sources of microorganisms on the dressed carcass [Dc; **(A)**], cold carcass [Cc; **(B)**] and the shoulder [S; **(C)**] samples generated by the SourceTracker software. The proportion (%) that each source represents on the selected meat sample is indicated in parentheses. Samples were collected from air (Ar), feces (Fc), saliva (Sa), feed (Fe), drain at evisceration (Dev), conveyor at evisceration (Cev), blood collection gutter (Gev), drain at cut-out (Dcu), conveyor at cut-out (Ccu).

A source tracking analysis was used to identify possible sources of microbial contamination on the carcasses and shoulder cuts. As shown in Figure 8A, most microorganisms found (as ASVs) in Dc samples did not likely originate from the farm since there were limited ASVs ascribed to them. Farm samples (Ar, Fc, Sa, and Fe) are the source of only 2.2% of the ASVs also found in Dc samples. The contamination sources remain mostly unknown (81%), but 14.4% came from the Dev samples. Figure 8B shows that overnight refrigeration after the initial blast chill did not change the microbiota of carcasses in a major way, since 89.9% of the ASVs were the same in Dc and Cc samples. Figure 8C indicates that most of the microorganisms detected in S samples likely originated from Cc (79.6%). The farm samples (Ar, Fc, Sa, and Fe) represented a total of 3.52% shared ASVs and most of the meat plant samples (Dev, Cev, Gev, Dcu, and Ccu) represent 9.39%.

## 4 Discussion

In this study, differences in the microbiota in samples taken from multiple steps along the value chain (from farm to meat cut-out area) were compared for two farms with different sanitary statuses. One aim was to measure the contribution of the farm and the meat plant to the microbiota found in the cuts of meat. The strength of our experimental design comes from the fact that the same animals that were raised on commercial farms were followed throughout the entire value chain.

Based on metataxonomic results, the concentration of SCFAs and metabolomic analysis of feces, the two farms were distinctive in terms of their microbiota. This is consistent with the observations made by the veterinarians who selected the farms. The hygiene conditions at farms have been shown to impact the microbiota in the gut of swine (Le Floc’h et al., 2014), their feed (Maciorowski et al., 2007) and the air (Duchaine et al., 2000). Results indicate that the bacterial genera identified in Ar and Fc samples were closely related as expected, since dried fecal particles are a major source of air contamination (Hamscher et al., 2003). Mechanical problems with the ventilation system in farm-H could explain some of the variation observed in the air microbiota. Inefficient ventilation is known to support higher concentrations of total microbes and higher percentages of fecal microbes in the air (Kim and Ko, 2019). These microbes, of which *Clostridium* are a large part of (Nehme et al., 2008; Song et al., 2021), clearly distinguished the two farms (Figure 1). High alpha diversity in the air in buildings where swine are raised has been linked to higher incidences of antibiotic resistance genes and opportunistic pathogens (Kumari et al., 2016; Yan et al., 2021). In our study, ASV counts in Ar samples were higher than those reported in the literature (Kraemer et al., 2019; Yan et al., 2021). The high observed/ChaO1 alpha diversity we observed may be due, in part, to the pooling of all samples at the sample inference step of the bioinformatic pipeline, which can increase sensitivity to rare ASVs.

Because the feed comes into contact with saliva, it is expected that their bacterial composition might overlap and change with the type of feed. A month prior to sampling, farm-L experienced a salmonellosis outbreak, necessitating a switched to mash feed to control the diarrhea caused by the outbreak (Lo Fo Wong et al., 2004; Mikkelsen Lene et al., 2004; Longpré et al., 2016; Lebel et al., 2017; O'Meara et al., 2020). Mash feed is known to induce higher levels of SCFA in feces, which in turn improve hematological profile of pigs and reduce ulceration, diarrhea levels, as well as *Salmonella* and *E. coli* shedding (Lo Fo Wong et al., 2004; Mikkelsen Lene et al., 2004; Longpré et al., 2016; Lebel et al., 2017; O'Meara et al., 2020). Our Lefse analysis confirmed that the mash feed promoted well-known SCFA-producing bacteria, likely causing the higher concentration of SCFAs in the feces of animals from farm-L. This may also have contributed to stabilizing the microbiota, resulting in an alpha diversity similar to that of the feces from farm-H. Mash feed is also known to harbor higher bacterial counts than traditional hot pelleted feed (Mikkelsen Lene et al., 2004; Paramithiotis et al., 2009; O'Meara et al., 2020). Hence, the type of feed was an important factor in determining the differences between the two farms in our study.

The meat plant was clean before any of the animals under study were processed, allowing us to assess the contamination that came from the animals. Environmental samples of various types have been reported to range from 2 to 6 Log_10_ of CFU/cm^2^ for TAM and from undetected to 5 Log_10_ of CFU/cm^2^ for EB (Warriner et al., 2002; Bridier et al., 2019; Maes et al., 2019). Our microbial counts for TAM and EB were below those values in Cev, Gev, Ccu, and Dcu samples. Only the Dev samples exhibited highly variable levels of contamination over time. This is consistent with the literature which suggests that processing plant drains are a source of contamination, notably through aerosol formation (Byrne et al., 2008), and they are a main contributor to the contamination variation and evolution of a processing plant over time (ICMSF, 2018).

Overall, contamination level of the meat plant was similar after the animals under study were processed for both farms. However, season is an important contributing factor of contamination in swine raising environments (Kumari et al., 2016) and its effect should be addressed over a longer period of time. Carcass (Dc and Cc; Figure 2) and drain samples (Dev and Dcu; Figure 3) had similar numbers of TAM and EB compared to the range reported in the literature (Spescha et al., 2006; Gill and Badoni, 2010; Zwirzitz et al., 2020), whereas Cev, Gev and Ccu samples (Figure 3) were lower (Warriner et al., 2002; Bridier et al., 2019; Zwirzitz et al., 2020). Total aerobic mesophilic, *Enterobacteriaceae* and coliform counts increased on the processing equipment over time (Warriner et al., 2002). The low counts observed may be explained by the fact that the animals examined were the first of the day to be slaughtered. They spent less than an hour on the processing line, which processed 500 animals per hour. Gev and Dcu samples had a significant increase in TAM after the animals were processed (Figure 3). This was not the case for the EB counts. Gev mainly collected blood and if swine were healthy, their blood contained a limited number of microbes (Hyun et al., 2021). This may explain why EB counts did not change over the short sampling period in Gev samples. Hot water flowed through Dcu during operation. Because enterobacteria do not form spores (Brenner and Farmer, 2015), they likely could not survive in those conditions. Cell counts suggest that contamination risks are similar for both farms. The slightly higher relative abundance of *Salmonella* in samples from farm-L might suggest otherwise, although only trace amounts were detected (0.036% relative abundance in Fe and 0.0045% on Cev).

Alpha diversity decreased in samples along the value chain, suggesting that the microbiota become less complex especially for Dc, Cc, and S samples. This progressive diversity reduction is partially explained by the heat treatment (scalding and singeing) that is part of processing the animal, and the subsequent dehairing and polishing procedures. These steps remove a large portion of the skin microbiota which could be recontaminated by the spreading of resident bacteria from the meat plant onto the carcasses (Gill and Bryant, 1993; Gill, 2000; Wheatley et al., 2014; Zwirzitz et al., 2020). In Zwirzitz et al. (2020), alpha diversity decreased between the arrival of animals at the slaughterhouse and singeing, increased again at the polishing step, and then decreased continuously until carcasses were shipped to a meat cutting facility. This indicates that polishing is a critical step in replacing the animal microbiota on the surface of the carcasses with resident bacteria from the slaughterhouse. Clearly, the processing steps prior to the overnight refrigeration warrant further investigation. In our study, this process could explain how the beta diversity for carcasses from both farms were not significantly different. The sample type and location (farm vs. evisceration area vs. cut-out area) significantly affected the structure of the microbiota community (Table 2).

There are indications that some of the bacteria from the farms were still present on the carcasses and the shoulder samples. *Escherichia/Shigella* were detected mainly in Sa and Fe samples at the farms and in Dev, Cev, and Gev samples at the evisceration area. The same *Escherichia/Shigella* ASV detected in Sa and Fc samples were also found in all meat plant samples (ASV168; Supplementary Table S2) and traces were detected in Cc and S samples*. Campylobacter,* the most common pathogen in the swine value chain (Farzan et al., 2010; Baer et al., 2013), was detected in Sa, Fe, and S samples. The data revealed that the ASVs detected in the S samples were not the same as those found in Sa, Fe, Sa, Dev, and Dcu (Supplementary Table S2) and were instead the same as Cev, Gev, and Ccu. This suggests that a portion of the detected *Campylobacter* might be from meat plant surfaces. Furthermore, *Carnobacterium* were detected almost exclusively at the cut-out location, indicating that these bacteria are from the meat plant. These bacteria are nearly absent on carcasses but were identified on S samples. Interestingly, this genus, along with many *Enterobacteriaceae* (Supplementary Figure S2; *Enterobacter, Citrobacter, Buttilauxella, Lelliottia* and *Kluyvera*), replaced *Lactobacillus*, *Moraxella* and *Staphylococcus* on S samples. This could explain why *Enterobacteriaceae* counts increased on the shoulders instead of following the general downward trend of the total aerobic mesophilic counts after carcass breakdown. Multiple studies (Gill and Bryant, 1993; Gill et al., 1999; Gill, 2000; Gill and Sofos, 2005) investigated the impact of carcass breakdown on meat microbiota. Multiple types of bacteria are transferred from the equipment, workers, etc. to the resulting meat cuts during the processing steps in the value chain, further replacing any bacteria from the farm. The source tracking analysis confirms that only 12.92% of the microbiota on S samples originated from the farms and the meat plant environment (Figure 8C).

Overall, 81% of the bacteria detected in the Dc samples were of unknown origin (Figure 8A) indicating that much remains to be investigated to understand the sources of contamination found in the final cuts of meat. Dehairing and polishing processes would be valuable steps to consider in determining sources of contamination (Gill, 2000). Once carcasses were blast chilled, the microbiota was less susceptible to variation between Dc, Cc and S samples; 89.9% of the bacteria found in the Cc samples came from the Dc and 79.6% of the bacteria found on S samples came from Cc. Refrigeration favors the development of a more psychrotrophic microbiota (Zwirzitz et al., 2020) and on S samples some cold-tolerant bacteria were detected, as the cut-out area was maintained at 7°C at all times (Figure 1; *Pseudomonas, Psychrobacter*; Juni, 2015; Palleroni, 2015).

## 5 Conclusion

The variability in the microbiota observed in samples along the value chain were more associated with the location (farm, evisceration area, cut-out area) and sample type rather than the farm of origin or the shipment week. The microbes identified on each farm were largely controlled by in-house operational procedures. This is evidenced by the fact that less than 4% of bacteria identified in the S samples, the last step in the value chain that was examined, were determined to be from the farms. In fact, a more homogeneous and less complex microbiota was observed as we moved forward along the value chain. However, it would be presumptuous to claim that meat plants completely decontaminate animals from their initial microbiota acquired at the farm regardless of their sanitary status since *Salmonella* was found in low relative abundance with Fe and Cev samples associated to farm-L. The results rather suggest that the microbial control that is presently achieved by farms makes it possible to produce pork products with adaquate microbiological quality. Nevertheless, a broader study that includes more farms with different sanitary statuses is needed to accurately determine the impact and magnitude of farm sanitary status on the final commercial pork products knowing that many factors such as season, feed form and composition, housing conditions, preslaughter management, etc., are also contributing factors.

## Data Availability

The datasets presented in this study can be found in online repositories. The names of the repository/repositories and accession number(s) can be found in the article/Supplementary Material.
