# Supplementary material for: Contribution of farms to the microbiota in the swine value chain
Source: Front Syst Biol. 2023 Jul 12;3:1183868. doi: 10.3389/fsysb.2023.1183868 (PMC12342041; doi:10.3389/fsysb.2023.1183868)
Supplement: Supplementary file 3 [file Table1.DOCX]

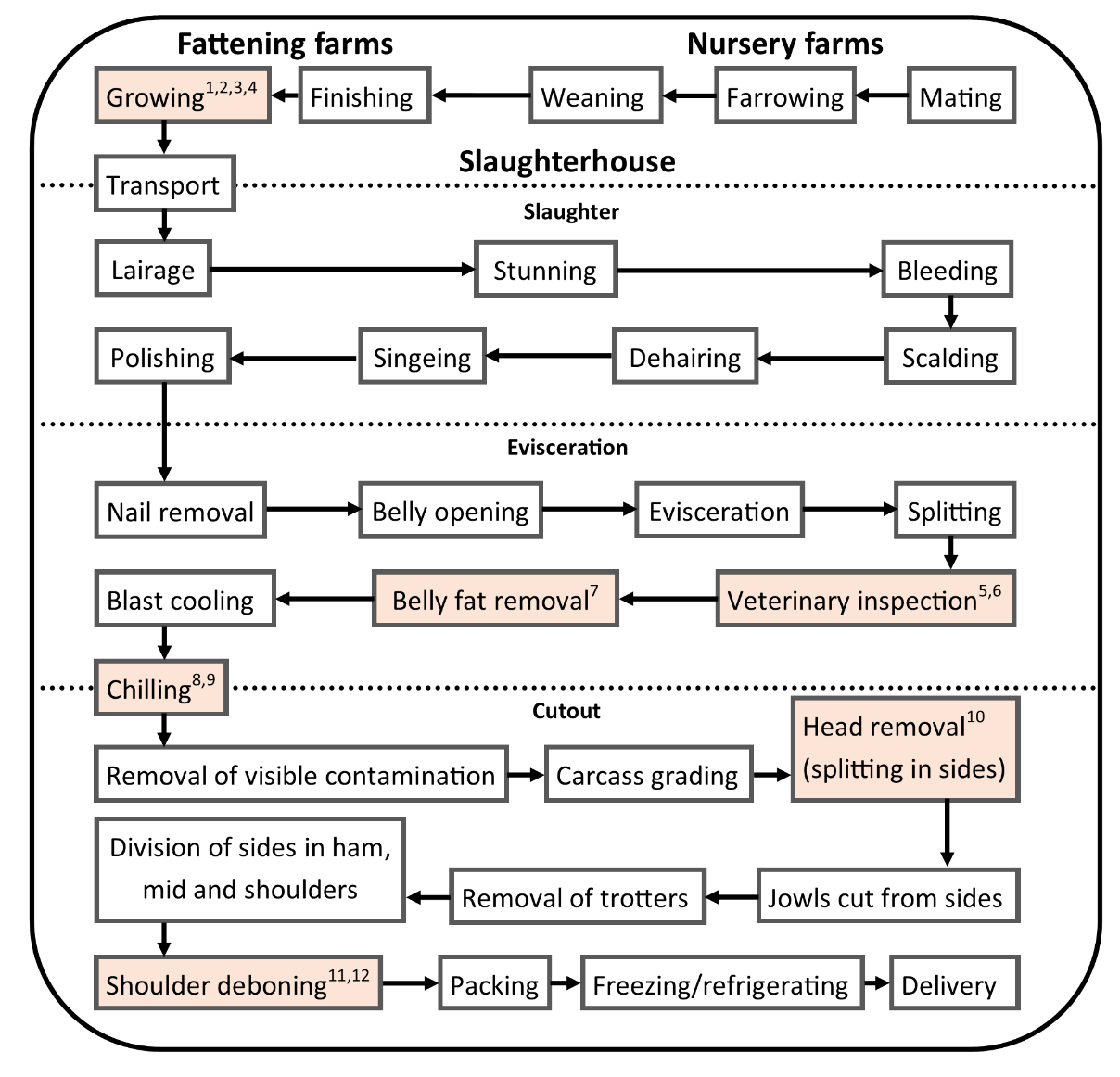


**FIGURE S1**

Flow chart of the location of various samples taken throughout the pork value chain at the selected farms and meat plant. Sample locations are highlighted in orange. Samples were collected from air (Ar; 1), feces (Fc; 2), saliva (Sa; 3), feed (Fe; 4), drain at evisceration (Dev; 5), conveyor at evisceration (Cev; 6), blood collection gutter (Gev; 7), dressed carcasses (Dc; 8), cold carcasses (Cc; 9), drain at cut-out (Dcu; 10), conveyor at cut-out (Ccu; 11), shoulder (S; 12).m


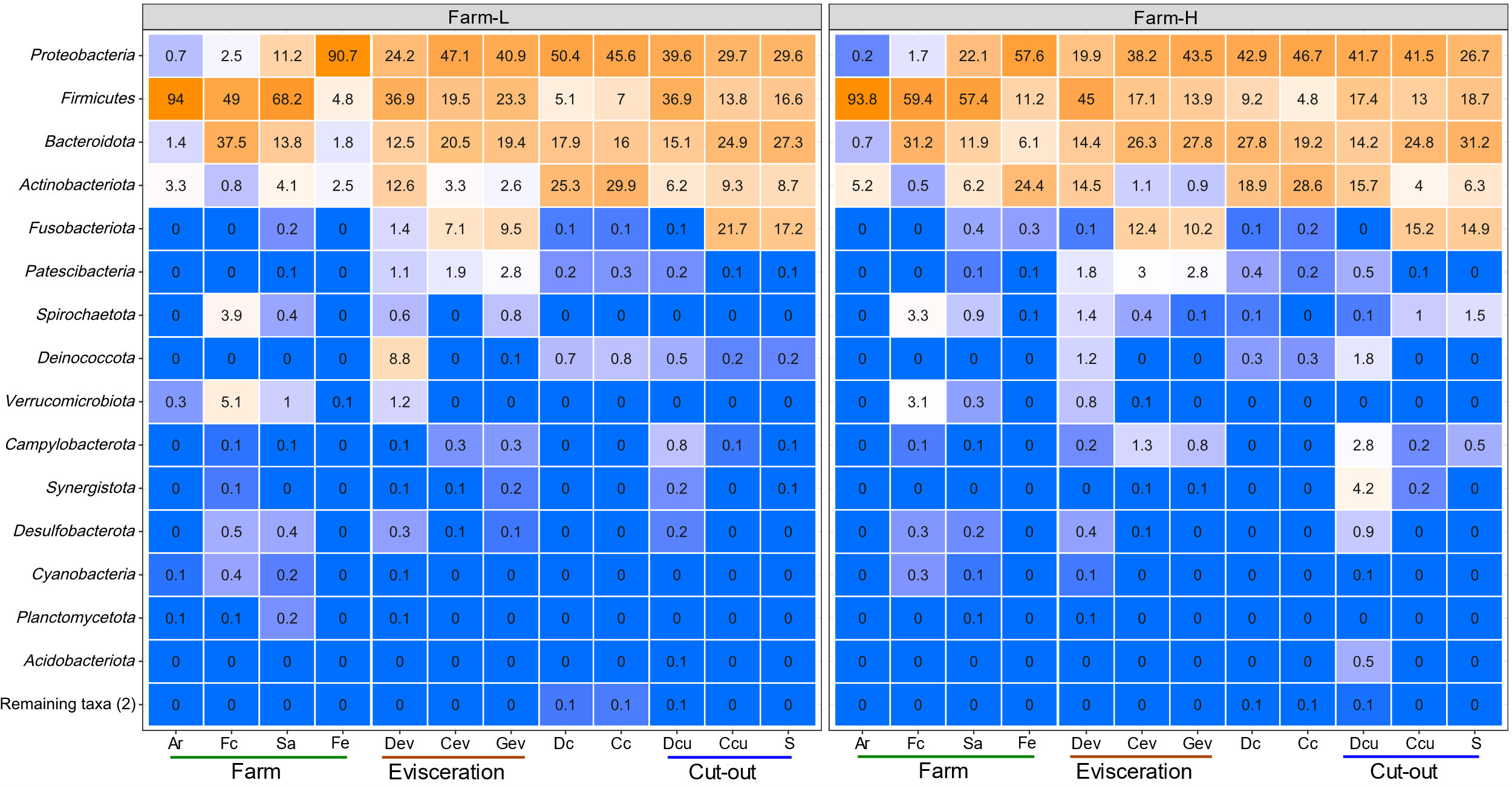


**FIGURE S2**

Top 15 phyla based on relative abundance (%) identified in each of the different sample types for both farms. Phyla were identified using the SILVA database. Colour gradients range from blue = 0% to orange = 100%. Samples were collected from air (Ar), feces (Fc), saliva (Sa), feed (Fe), drain at evisceration (Dev), conveyor at evisceration (Cev), blood collection gutter (Gev), dressed carcasses (Dc), cold carcasses (Cc), drain at cut-out (Dcu), conveyor at cut-out (Ccu) and shoulder (S). Farm-L with a lower and farm-H with a higher sanitary status, respectively.

**A**

**
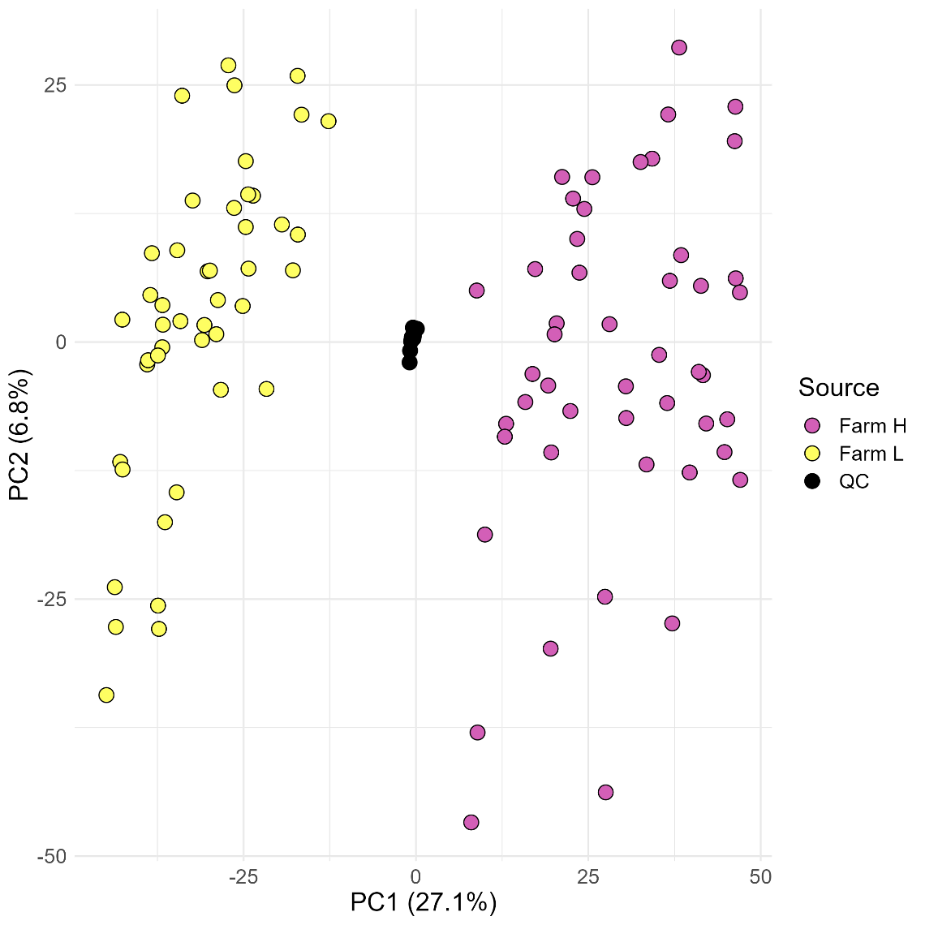
**

**B**

**
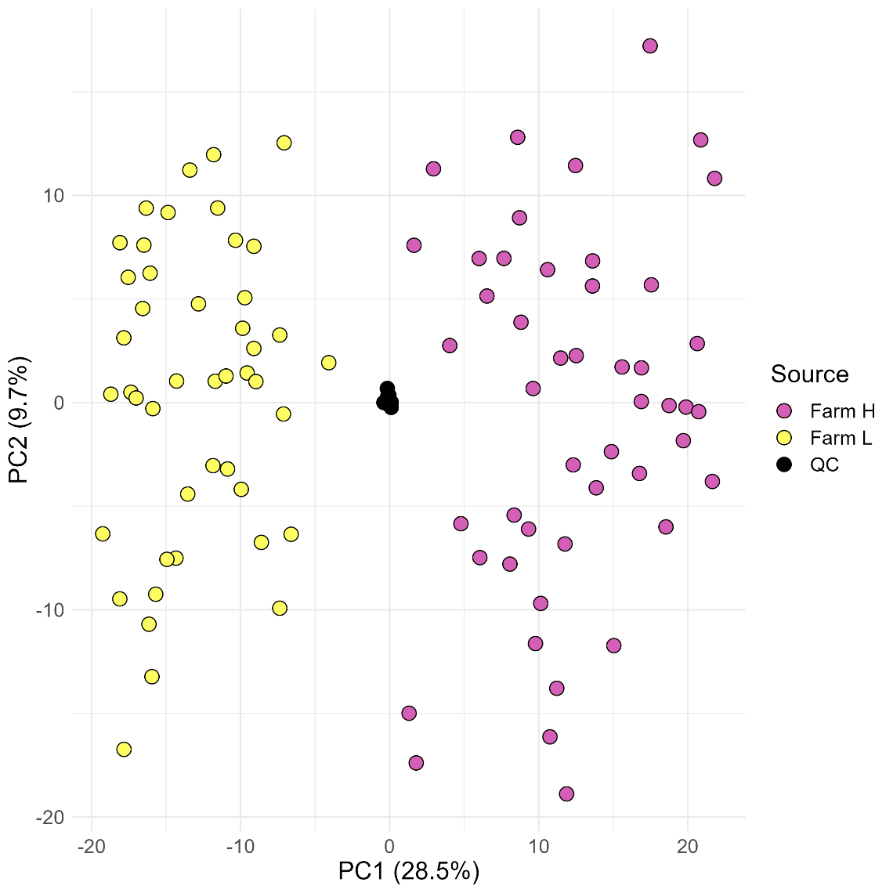
**

**FIGURE S3**

Principal Component Analysis (PCA) of every ion generated by positive (A) and negative (B) ionization from swine feces samples from farm-L (yellow) or farm-H (pink), with lower and higher sanitary statuses, respectively. The blue points in the center of the graph are the QC pools.


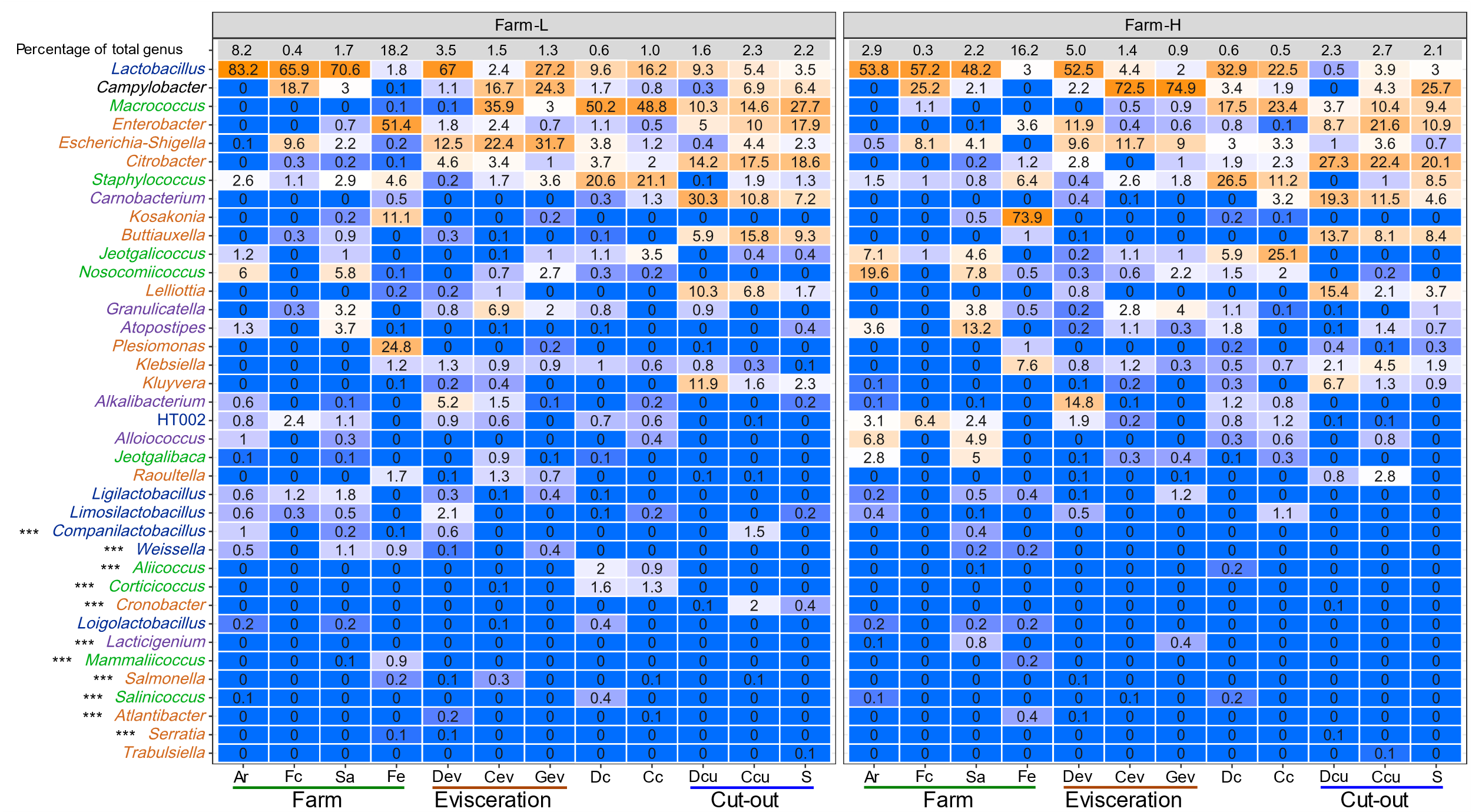


**FIGURE S4**

Relative abundance (%) of genera belonging to the *Campylobacteraceae* (black), *Carnobacteriaceae* (purple), *Enterobacteriaceae* (brown), *Lactobacillaceae* (blue) and *Staphylococcaceae* (green) families compared to one another and calculated for each of the sample types for both farms. The top row indicates the percentage that these genera represented in the total microbiota of each sample type. Colour gradients range from blue = 0% to orange = 100%. Samples were collected from air (Ar), feces (Fc), saliva (Sa), feed (Fe), drain at evisceration (Dev), conveyor at evisceration (Cev), blood collection gutter (Gev), dressed carcasses (Dc), cold carcasses (Cc), drain at cut-out (Dcu), conveyor at cut-out (Ccu) and shoulder (S). Farm-L with a lower and farm-H with a higher sanitary status, respectively. Significant difference between farm using ANCOMBC methodology is highlighted with *: *P*≤0.05, **: *P*≤0.01, ***: *P*≤ 0.001.


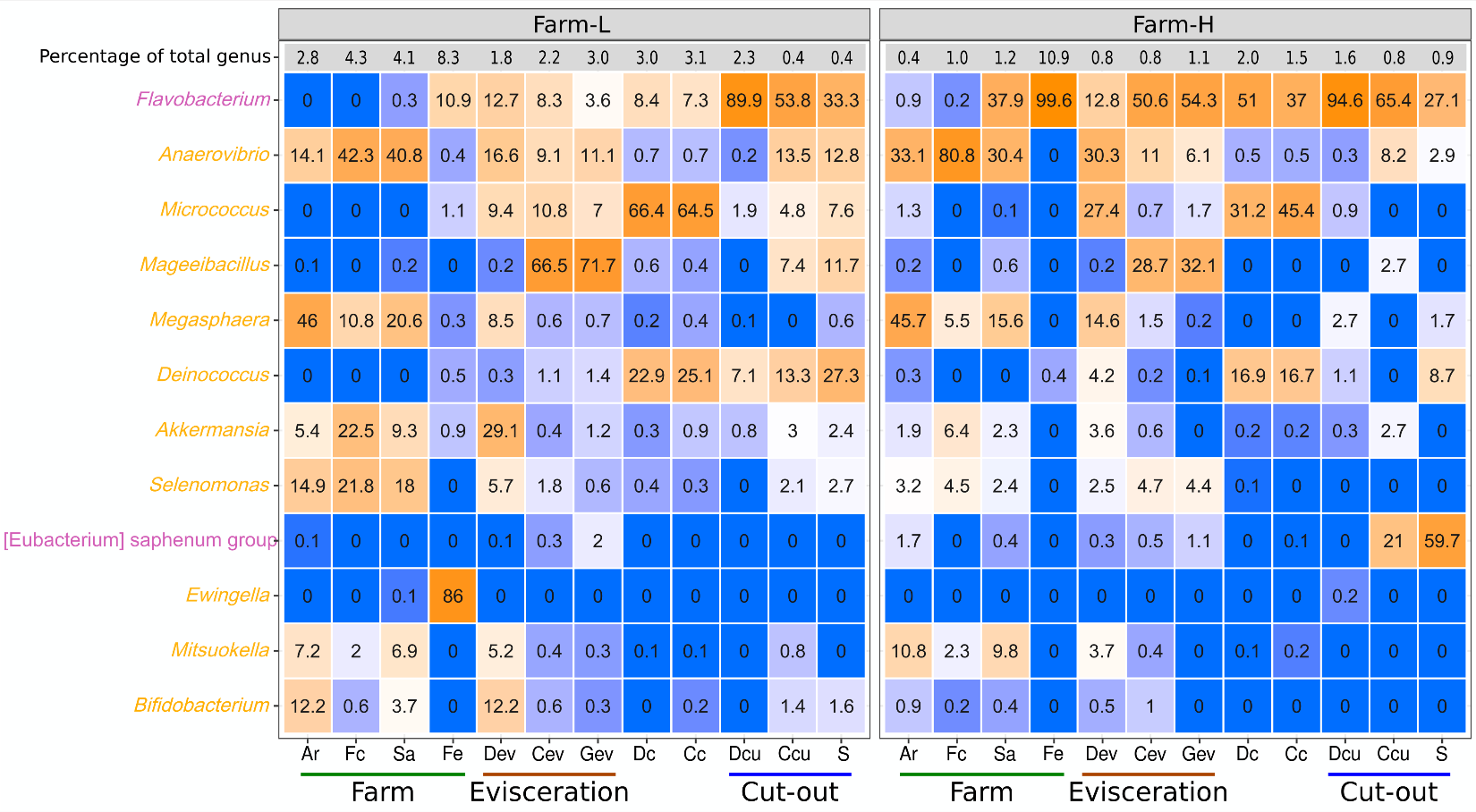


**FIGURE S5**

Relative abundance (%) of genera identified by LEfSe analysis as biomarkers of farm-L (yellow) or farm-H (pink) with lower and higher sanitary statuses, respectively, for each of the sample type. Percentage was calculated in reference to one another. The top row indicates the percentage that these genera represented in the total microbiota of each sample type. Colour gradients range from blue = 0% to orange = 100%. Samples were collected from air (Ar), feces (Fc), saliva (Sa), feed (Fe), drain at evisceration (Dev), conveyor at evisceration (Cev), blood collection gutter (Gev), dressed carcasses (Dc), cold carcasses (Cc), drain at cut-out (Dcu), conveyor at cut-out (Ccu) and shoulder (S).
